# Multifunctional cellulase catalysis targeted by fusion to different carbohydrate-binding modules

**DOI:** 10.1186/s13068-015-0402-0

**Published:** 2015-12-21

**Authors:** Johnnie A. Walker, Taichi E. Takasuka, Kai Deng, Christopher M. Bianchetti, Hannah S. Udell, Ben M. Prom, Hyunkee Kim, Paul D. Adams, Trent R. Northen, Brian G. Fox

**Affiliations:** US Department of Energy Great Lakes Bioenergy Research Center, University of Wisconsin-Madison, Madison, WI 53706 USA; Department of Biochemistry, University of Wisconsin-Madison, Madison, WI 53706 USA; Research Faculty of Agriculture, Hokkaido University, Sapporo, 060-8589 Japan; US Department of Energy Joint BioEnergy Institute, Emeryville, CA 94608 USA; Sandia National Laboratories, Livermore, CA 94551 USA; Department of Chemistry, University of Wisconsin-Oshkosh, Oshkosh, WI 54901 USA; Lawrence Berkeley National Laboratory, Berkeley, CA 94720 USA; Department of Bioengineering, University of California, Berkeley, CA 94720 USA

**Keywords:** Cellulase, Xylanase, Hemicellulase, Mannanase, Carbohydrate binding module, *Ruminoclostridium thermocellum*, Enzyme engineering, Biofuels, Mass spectrometry, Kinetic analysis

## Abstract

**Background:**

Carbohydrate binding modules (CBMs) bind polysaccharides and help target glycoside hydrolases catalytic domains to their appropriate carbohydrate substrates. To better understand how CBMs can improve cellulolytic enzyme reactivity, representatives from each of the 18 families of CBM found in *Ruminoclostridiumthermocellum* were fused to the multifunctional GH5 catalytic domain of CelE (Cthe_0797, CelEcc), which can hydrolyze numerous types of polysaccharides including cellulose, mannan, and xylan. Since CelE is a cellulosomal enzyme, none of these fusions to a CBM previously existed.

**Results:**

CelEcc_CBM fusions were assayed for their ability to hydrolyze cellulose, lichenan, xylan, and mannan. Several CelEcc_CBM fusions showed enhanced hydrolytic activity with different substrates relative to the fusion to CBM3a from the cellulosome scaffoldin, which has high affinity for binding to crystalline cellulose. Additional binding studies and quantitative catalysis studies using nanostructure-initiator mass spectrometry (NIMS) were carried out with the CBM3a, CBM6, CBM30, and CBM44 fusion enzymes. In general, and consistent with observations of others, enhanced enzyme reactivity was correlated with moderate binding affinity of the CBM. Numerical analysis of reaction time courses showed that CelEcc_CBM44, a combination of a multifunctional enzyme domain with a CBM having broad binding specificity, gave the fastest rates for hydrolysis of both the hexose and pentose fractions of ionic-liquid pretreated switchgrass.

**Conclusion:**

We have shown that fusions of different CBMs to a single multifunctional GH5 catalytic domain can increase its rate of reaction with different pure polysaccharides and with pretreated biomass. This fusion approach, incorporating domains with broad specificity for binding and catalysis, provides a new avenue to improve reactivity of simple combinations of enzymes within the complexity of plant biomass.

**Electronic supplementary material:**

The online version of this article (doi:10.1186/s13068-015-0402-0) contains supplementary material, which is available to authorized users.

## Background

The sustainable and economically viable production of biocommodities from cellulosic biomass poses a significant challenge due to the recalcitrant nature of plant cell walls [1–3]. Cellulose, the primary source of fermentable sugars [4–6], is surrounded by hemicellulose [7, 8], a heterogeneous assemblage of different polysaccharides derived from xylose, arabinose, galacturonate, fucose, mannose and other sugars, and lignin, a highly variable aromatic polymer derived from phenylalanine [9, 10]. The cellulose polymer is composed of pure β-1,4-linked glucose present in different crystalline allomorphs depending on the species origin and the handling of the material [11–15]. Intra-chain hydrogen-bonding creates various crystalline and recalcitrant structures. Hemicellulose is assembled from a variable combination of sugar backbones and may have a variety of branching structures and species-specific variations [8, 16, 17]. For example, xyloglucan consists of a β-1,4-linked glucose with partial backbone acetylation and O6 branches containing xylose, galactose, and fucose [8], while glucuronoarabinoxylan consists of a β-1,4-linked xylose with partial backbone acetylation and O2 and O3 branches containing arabinose and glucuronate [8]. Ferulate esters also serve to crosslink the arabinoxylan branches to lignin [8]. Altogether, the complex matrix of cellulose, hemicellulose, and lignin is a primary impediment to the high-yield enzymatic deconstruction of biomass [6, 18, 19]. In order to achieve the inherent potential of a renewable biocommodities industry based on sugars derived from cellulosic biomass, improvements in many technologies including chemical pretreatment, enzyme hydrolysis, and microbial fermentation are still needed [5, 19–21].

To overcome the recalcitrance of plant cell walls, microorganisms produce a multitude of cellulases, hemicellulases, lytic polysaccharide monooxygenases (LPMOs), and other enzymes [16, 21–23]. Cellulases are divided into endoglucanases, exoglucanases, and β-glucosidases that synergistically convert cellulose into glucose [6, 22, 24, 25]. Hemicellulases such as xylanases and mannanases release fermentable sugars from xylan and mannan, respectively [16, 26, 27]. The LPMOs (AA9 and AA10, formerly known as GH61 and CBM33 [28]), are found in aerobic fungi and microbes, and introduce oxidative nicks into polysaccharides [29–36]. These enzymes are typically composed of a catalytic domain and a carbohydrate binding module (CBM) that targets binding of the catalytic domain to a specific substrate [37].

To date, more than 48,000 CBM sequences have been classified into 71 CBM families based on sequence similarity, and the structures of 271 representative CBMs have been reported (http://www.cazy.org) [38]. The many CBM families contain members that bind to the various polysaccharides that occur in nature [39, 40]. Three types of CBMs have been identified based on their structures and ability to influence the function of associated catalytic domains [37]. Type A CBMs interact with the planar surfaces of crystalline polysaccharides, such as cellulose, through interactions between aromatic amino acid side chains of Trp, Tyr, and Phe [41, 42] and the polysaccharide. Type B CBMs have an open cleft that can bind polysaccharides found in amorphous regions of cellulose and hemicellulose [43–46]. Type C CBMs are suggested to bind short soluble oligosaccharides [37]. Therefore, different types of CBMs can target an attached catalytic domain to a particular substrate, and by doing so, have profound effects on the catalytic rates of the attached enzyme [47–52].

*Ruminoclostridium thermocellum* (formerly *Clostridium thermocellum*), a thermophilic, cellulolytic, and ethanologenic anaerobe, extracts nutrients from lignocellulosic biomass by producing a multi-enzyme complex called a cellulosome [53–57]. This complex is formed by the recruitment of enzymes to the scaffoldin protein, CipA (Cthe_3077), as a result of high-affinity interactions of dockerin and cohesin domains. Both the scaffoldin and recruited enzymes contain CBMs that attach to insoluble substrates. For example, CBM3a is an integral domain of scaffoldin CipA, and it helps to localize the cellulosome to the surface of crystalline cellulose to promote efficient hydrolysis [41]. In addition, many cellulosomal enzymes possess their own CBMs that localize them to additional substrates in close proximity to cellulose. *R. thermocellum* is thus an invaluable source of both enzyme catalytic domains and CBMs for studies to identify unique pairs with enhanced reactivity.

The use of native and engineered enzymes has the potential to reduce the cost of biofuel production [58]. Current fungal cocktails used for biomass hydrolysis are complex and might contain 50 or more different polypeptides [59]. A number of approaches are being considered that could improve the performance of enzyme mixtures in biomass deconstruction, including (1) elimination of redundant or nonfunctional proteins from the mixture; (2) stabilization of key enzymes from nonspecific irreversible adsorption, proteolytic, thermal, and other types of inactivation; and (3) substitution of enzymes with different binding properties, *k*_cat_, or other catalytic properties better matched to the conditions of the desired application. Recently, we reported that CelE [60], a single broad specificity glycoside hydrolase family 5 (GH5) domain from *R. thermocellum*, is able to hydrolyze cellulose, xylan, and mannan, the three major polysaccharides found in the plant cell wall, and so could potentially replace or augment more strictly specific cellulose-, xylan-, or mannan-degrading enzymes in a hydrolysis reaction. We also showed that the fusion of the CelE catalytic core/domain (CelEcc) to CBM3a was highly reactive on pretreated biomass [61]. With this positive result, it was reasonable to consider whether other CBM domains might enhance this broad reactivity. Indeed, the ability to target enzymes toward different polysaccharide constituents of plant biomass via engineered fusion to CBMs with different binding specificities is an intriguing [51, 62–68], albeit not fully explored, aspect of glycoside hydrolase engineering.

In this paper, we report a combinatorial evaluation of the ability of representatives from each of the 18 CBM families found in *R. thermocellum* to modulate enzyme function of a single multifunctional enzyme, CelE, from the same organism. Following earlier studies where fluorescent proteins have been appended to CBMs and other proteins to better understand their binding properties [68–75], GFP_CBM fusions were used to study CBM binding. Enzyme_CBM fusions were then used to study effects on catalytic activity with purified polysaccharides and with ionic liquid pretreated switchgrass (IL-SG), a model bioenergy substrate containing amorphous cellulose and retaining a high fraction of hemicellulose [76, 77]. Results show fusions of different CBMs to CelE gave enhancement of both rates and yields in hydrolysis with different purified polysaccharide substrates and also with IL-SG. The best improvements in reactivity for the same catalytic domain (~4×) were correlated with broad specificity and moderate affinity of CBM binding.

## Results

### CBMs from *R. thermocellum*

Thirty-nine CBMs from *R. thermocellum* ATCC 27405 were selected for study (Additional file 1: Table S1), including nine representatives from family CBM3, seven from CBM4, two from CBM9, five from CBM22, three from CBM35, and one each from CBM6, CBM11, CBM13, CBM16, CBM25, CBM30, CBM32, CBM34, CBM42, CBM44, CBM48, CBM50, and CBM54 (Additional file 1: Table S1). In order to test all of the CBM classes encoded in the *R. thermocellum* genome, at least one sequence was selected from each family. When multiple sequences were found in the CBM family (i.e., there are 24 genes encoding a CBM3 domain in *R. thermocellum*), several sequences were selected to test for their functions.

**Fig. 1:**
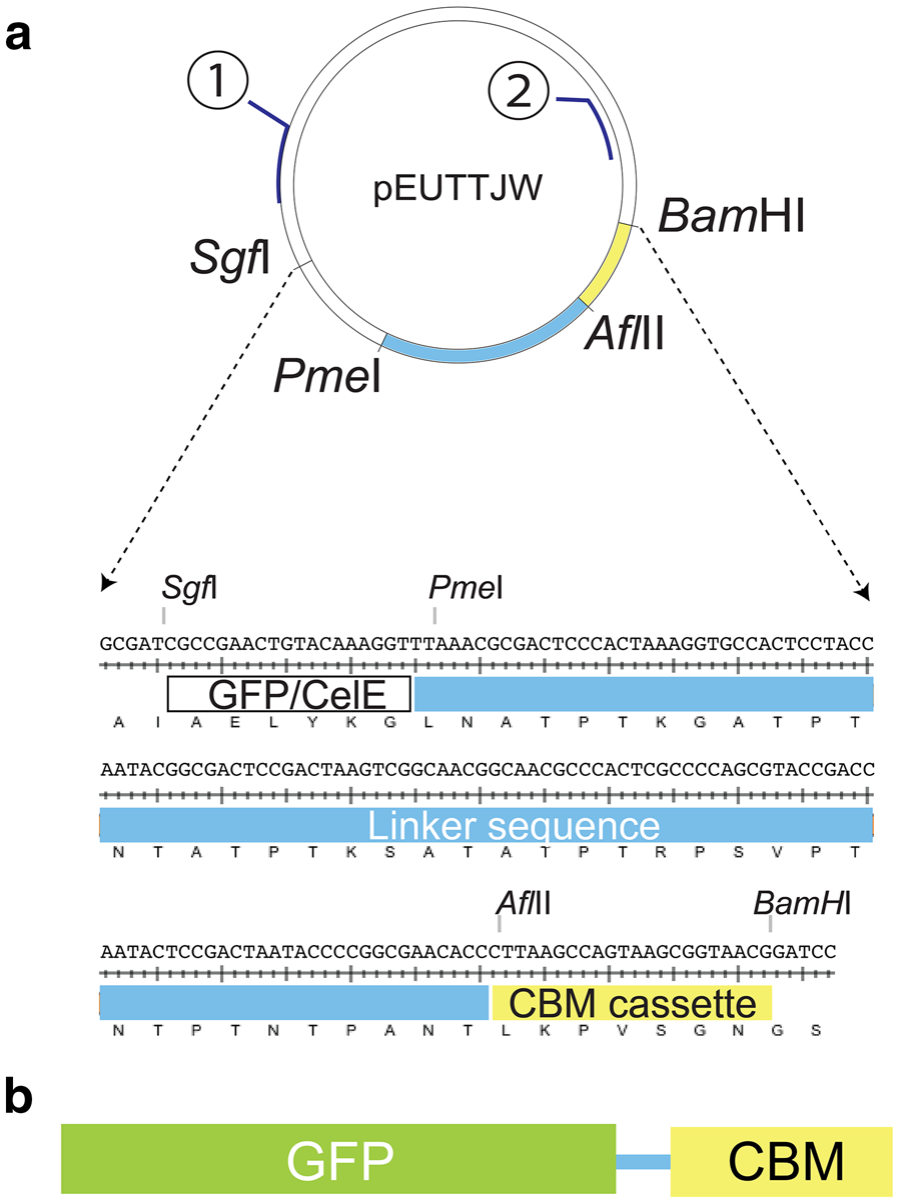
Schematics of the plasmid and fusion proteins used in this work. **a** Schematic of the plasmid and nucleotides in the functional region of pEUTTJW used to create fused gene sequences for cell-free protein translation. Locations of flanking primer pair used to transfer an assembled fusion protein into pVP67K for expression in *E. coli* are shown as *circles* *1* and *2* (*blue* *lines*). **b** Schematic of the domain structures of expressed protein consisting of either GFP (*green*) or CelE (*purple*), followed by the linker (*blue*) and the CBM domain (*yellow*)

**Fig. 2:**
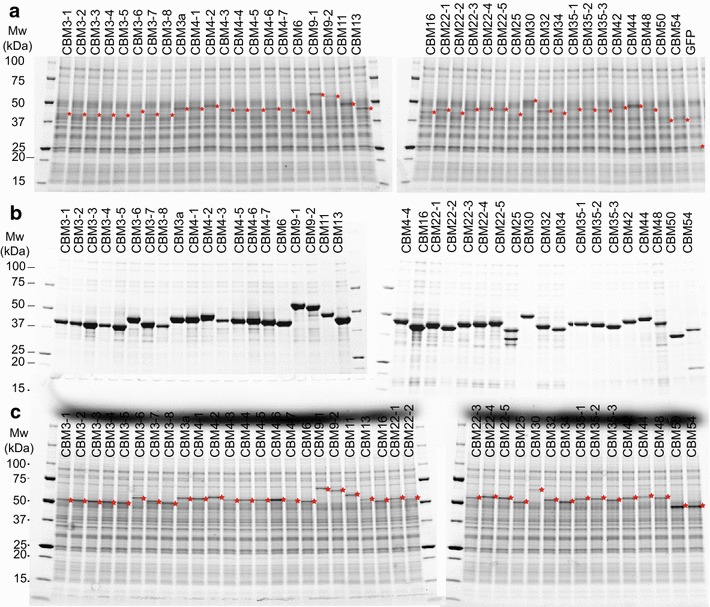
SDS PAGE analysis of proteins and enzymes. **a** Expression of GFP_CBM constructs in the cell-free translation reaction. **b** GFP_CBMs after Ni-IMAC purification from the cell-free translation reaction. These samples were used in experiments of Figs. 3 and 5. **c** Expression profile of CelEcc_CBM constructs in the cell-free translation reaction. These samples were used in experiments of Fig. 4. *Red* *stars* indicate the position of the synthesized proteins. Molecular weights calculated from the gene sequence and estimated protein concentrations are provided in Additional file 1: Tables S1, S4

The plasmid pEUTTJW (Fig. 1) was designed to contain four unique restriction enzyme recognition sites, *Sgf*I, *Pme*I, *Afl*II, and *Bam*HI, which allowed PCR-amplified DNA sequences (Additional file 1: Tables S2, S3) to be swapped into either the catalytic domain, linker, or CBM positions. By means of *Afl*II and *Bam*HI restriction enzymes, the set of GFP_CBM plasmids was constructed. Subsequently, *Sgf*I and *Pme*I restriction enzymes were used to create the corresponding CelEcc_CBM plasmids. All genes were successfully cloned, sequence-verified, and translated into protein products using wheat germ cell-free translation (Fig. 2; Additional file 1: Table S4).

### Soluble polysaccharide binding

**Fig. 3:**
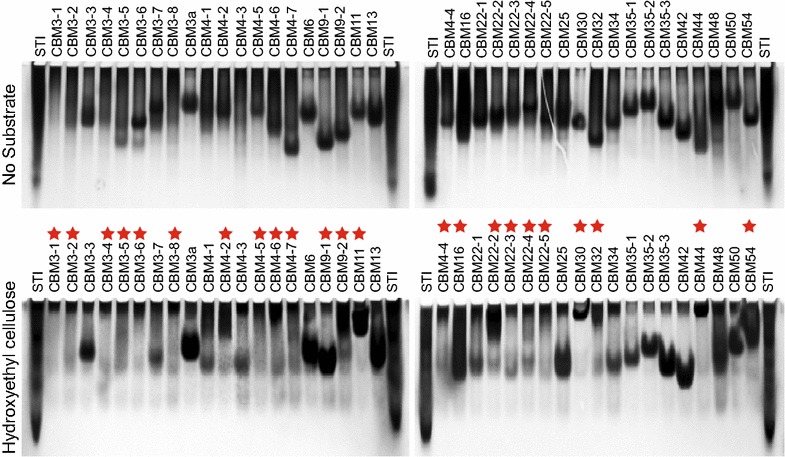
Affinity gel electrophoresis characterization of GFP_CBM binding to hydroxyethyl cellulose. GFP_CBMs purified from the translation reaction using Ni-IMAC were used in these experiments. Binding was detected as a difference in migration for the “No substrate” gel compared to the hydroxyethyl cellulose gel. *Red* *stars* indicate GFP_CBM fusions assigned to have altered migration, and so are inferred to have binding properties. Images of other electrophoresis gels containing lichenan, galactomannan, beechwood xylan, and arabinoxylan are provided in Additional file 2: Figure S1. Binding assignments made from all affinity gel electrophoresis studies are summarized in Table 1. Soybean trypsin inhibitor (STI) was used as a control

To determine the binding specificity of the *R. thermocellum* CBMs, we performed affinity gel electrophoresis with GFP_CBMs and soluble polysaccharides including hydroxyethylcellulose (HEC), icelandic moss lichenan, carob galactomannan, beechwood xylan, and wheat flour arabinoxylan. GFP_CBM binding was evaluated by calculating *R*_r_ from gels prepared with and without substrate. Most of the constructs that bound soluble substrates had *R*_r_ values less than 0.75. *R*_r_ values are listed in Additional file 1: Table S5. Twenty-eight GFP_CBMs interacted with at least one of the substrates tested; 23 and 17 GFP_CBMs were assigned to bind to either HEC or lichenan, respectively (Fig. 3; Table 1; Additional file 2: Figure S1). Among all CBMs tested, CBM44 showed the broadest binding specificity.

**Table 1:** Qualitative determination of binding specificities of GFP_CBMs to soluble substrates

GFP_CBM	HEC	Lichenan	Galactomannan	Beechwood xylan	Arabinoxylan
3-1	B	B	–	–	–
3-2	B	B	–	–	–
3-4	B	B	–	–	–
3-5	B	–	–	–	–
3-6	B	B	–	–	–
3-8	B	B	–	–	–
4-2	B	–	–	–	–
4-3	–	B	–	–	–
4-4	B	–	–	–	–
4-5	B	B	–	–	–
4-6	B	B	–	–	–
4-7	B	B	–	–	–
6	–	–	–	B	B
9-1	B	–	–	–	–
9-2	B	B	–	–	–
11	B	B	–	–	–
16	B	–	–	–	–
22-2	B	B	–	–	–
22-3	B	–	–	–	–
22-4	B	–	–	–	–
22-5	B	B	–	–	–
30	B	–	–	–	–
32	B	B	–	–	–
34	–	B	–	–	–
35-2	–	–	B	–	–
42	–	–	–	–	B
44	B	B	B	B	B
54	B	B	–	–	–

“B” indicates binding was detected by affinity gel electrophoresis; “–” indicates binding was not detected. Estimated Rr values for all CBMs tested in this study are shown in Additional file 1: Table S5

### Insoluble polysaccharide binding

Insoluble pull-down assays using GFP_CBMs were carried out with Avicel, phosphoric acid-swollen cellulose (PASC), birchwood xylan, 1,4-β-d-mannan, AFEX-SG, and IL-SG (Additional file 1: Table S6). Sixteen GFP_CBM constructs were detected to bind to one or more insoluble substrates according to the criterion of PF % of 10 % or greater (see Eqs. 2 and 3, “Methods”), and these are reported in Table 2. No binding was detected for 23 of the GFP_CBM constructs using the pull-down assay, and so are not included in Table 2.

**Table 2:** Qualitative determination of binding specificities of GFP_CBMs to insoluble substrates

GFP_CBM	Avicel	PASC	Mannan	Xylan	AFEX-SG	IL-SG
3-3	–	–	B	–	–	–
3a	B	B	–	–	B	B
4-5	–	B	–	–	–	–
4-6	–	B	–	–	–	–
6	–	–	B	–	–	–
9-2	–	B	–	–	–	B
11	–	B	B	–	B	–
16	B	B	B	–	–	–
22-2	–	–	–	B	B	–
22-3	–	–	–	–	B	–
25	–	–	–	B	B	–
30	–	B	–	–	–	–
35-1	–	–	–	B	B	–
35-2	–	–	–	–	B	B
35-3	–	B	–	B	B	–
50	–	–	–	B	–	–

“B” indicates binding was detected by pull-down assay; “–” indicates binding was not detected. Estimated PF %’s for all CBMs tested in this study are shown in Additional file 1: Table S6

### Catalytic properties of CelEcc_CBM

**Fig. 4:**
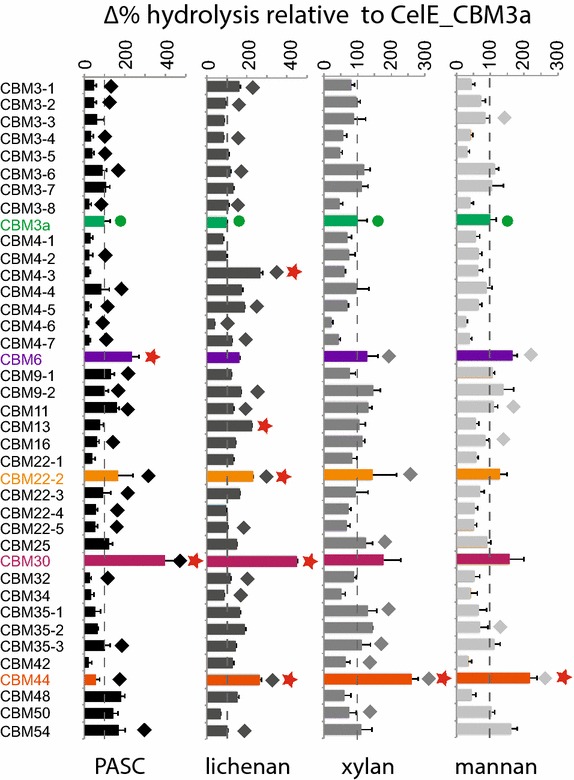
Hydrolytic activity of CelEcc_CBMs with purified polysaccharides. CelEcc_CBMs prepared using cell-free translation and tested, without purification from the translation reaction, for hydrolysis of phosphoric acid-swollen cellulose (PASC), lichenan, beechwood xylan, and mannan. A *red star* indicates a CelEcc_CBM hybrid that had a 200 % or more increase in activity relative to starting benchmark CelEcc_CBM3a (*green* *filled circle*). *Diamonds* indicate GFP_CBMs that bound to the indicated substrate according to binding experiments. Results with CBM3a, CBM6, CBM22-2, CBM30, and CBM44 are colored *green*, *purple*, *yellow*, *magenta,* and *orange*, respectively. Subsequent experiments focused on these five CBMs. *Error bars* indicate ±1 standard deviation from three independent experiments

Each CBM used in the binding assays was fused to the C-terminus of the GH5 catalytic domain of CelE (Cthe_0797), a multifunctional endoglucanase from *R. thermocellum* that can hydrolyze β-1,4-linkages in cellulose, xylan, mannan, and other polysaccharides [60, 61]. This breadth of activity provided an opportunity to study the abilities of different CBMs to target a single catalytic domain to different substrates. CelEcc_CBM3a served as the starting benchmark (Fig. 4, green bars and circles). CelEcc_CBM variants were tested for hydrolysis of PASC, icelandic moss lichenan, birchwood xylan, and 1,4-β-d-mannan (Fig. 4). CelEcc_CBM6 (purple bar) and CelEcc_CBM30 (magenta bar) displayed greater than a twofold increase in specific activity relative to CelEcc_CBM3a with PASC (indicated by a red star). For reactions with lichenan, CelEcc_CBM4-3 (red star), CelEcc_CBM13 (red star), CelEcc_CBM22-2 (yellow bar and red star), CelEcc_CBM30 (magenta bar and red star), and CelEcc_CBM44 (orange bar and red star) showed greater than twofold increase in hydrolytic activity relative to CelEcc_CBM3a. For reactions with xylan and mannan, CelEcc_CBM44 (orange bar and red star) showed greater than twofold increase in hydrolytic activity relative to CelEcc_CBM3a.

### Binding-affinity measurements

**Fig. 5:**
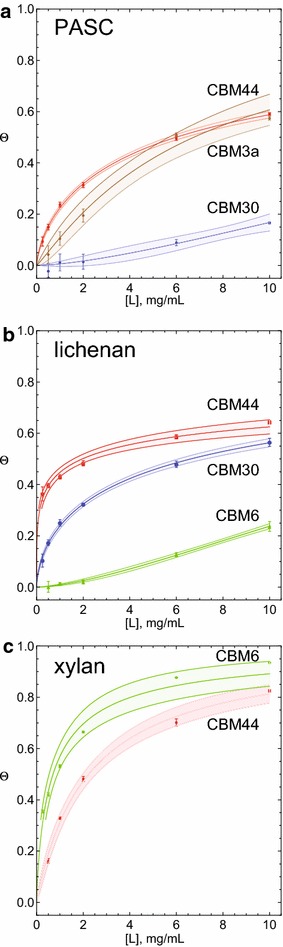
Binding-affinity plots for GFP_CBM fusions. GFP_CBMs used in this experiment were expressed in *E. coli* and purified as described in “Methods”. The fraction bound (*y*-axis) versus substrate concentration (*x*-axis) are shown for three different insoluble substrates. The *plots* were used to determine dissociation constants with the binding model given in Eq 6. *Shaded regions* around the plotted affinity curve are the mean prediction bands at the 90 % confidence level. **a** PASC plot and data fitting (GFP_CBM3a, *brown*; GFP_CBM30, *blue*; GFP_CBM44, *red*). **b** Lichenan plot and data fitting (GFP_CBM6, *green*; GFP_CBM30, *blue*; GFP_CBM44, *red*). **c** Xylan plot and data fitting (GFP_CBM6, *green*; GFP_CBM44, *red*)

Owing to results from the binding capability and enhancement of catalytic function when fused to CelE, we further studied the binding properties of four CBMs: CBM3a; CBM6; CBM30; and CBM44. Binding-affinity constants (*K*) for *E. coli*-expressed and -purified GFP_CBMs were calculated with PASC, icelandic moss lichenan, and oat spelt xylan (Fig. 5; Table 3). For PASC, GFP_CBM3a, GFP_CBM30, and GFP_CBM44 were determined to have *K*- and *c*-values of 8.26, 161.04, and 3.46 mg/mL; and 1.11, 1.51, and 0.69, respectively, while the *K-* and *c*-values of GFP_CBM6 for PASC could not be determined due to low affinity. With lichenan, GFP_CBM6, GFP_CBM30, and GFP_CBM44 had calculated *K*- and *c*-values of 110.44, 3.19, and 1.23 mg/mL, and 1.54, 0.61, and 0.31, respectively. GFP_CBM6 and GFP_CBM44 had *K*- and *c*-values of 0.76 and 2.22 mg/mL, and 0.79 and 0.99 for xylan, respectively. *K*- and *c*-values could not be ascertained for GFP_CBM3a with lichenan and xylan, and GFP_CBM30 with xylan. Of note, none of the four CBMs selected for these studies had a sufficiently high affinity for 1,4-β-d-mannan to be determined in these experiments.

**Table 3:** Dissociation and binding-interaction constants of GFP_CBMs constructs with insoluble polysaccharides

GFP_CBM	PASC			Lichenan			Xylan		
	*K*	*c*	*R* ^2^	*K*	*c*	*R* ^2^	*K*	*c*	*R* ^2^
3	8.26	1.11	0.9951	–	–	–	–	–	–
6	–	–	–	110.44	1.54	0.9992	0.76	0.79	0.9971
30	161.04	1.51	0.9825	3.19	0.61	0.9995	–	–	–
44	3.46	0.69	0.9997	1.23	0.31	0.9991	2.22	0.99	0.9989

“–” indicates binding affinity could not be determined

### Catalysis with IL-SG

**Fig. 6:**
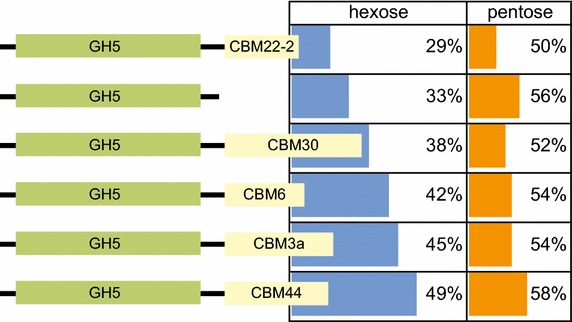
Domain structures/relative sizes of CelEcc_CBM hybrids and reaction with pretreated biomass. CelEcc_CBMs used in this experiment were expressed in *E. coli* and purified as described in “Methods”. The linker is from the CipA scaffoldin of the *R. thermocellum* cellulosome (amino acids 323–364 of Cthe_3077). Enzyme information is aligned with the yield of total soluble hexose and pentose products detected by quantitative NIMS analysis [61] after 24-h reaction in 50 mM phosphate, pH 6.0, at 60 °C. All reactions contained 0.32 µmol of enzyme active sites and 1 mg of ionic liquid pretreated switchgrass (IL-SG). The fractional sugar content of IL-SG (1 mg) was glucose (0.47 mg); xylose (0.18 mg), arabinose (0.03 mg), other sugars, lignin, and ash (0.32 mg). Percentage yields are based on these values and the NIMS product quantitation

CelEcc, CelEcc_CBM3a, CelEcc_CBM6, CelEcc_CBM30 and CelEcc_CBM44 were expressed in *E. coli* and purified to homogeneity. Equimolar active site concentrations (0.32 nmol) of these enzymes were reacted with IL-SG and the time course of sugar release was analyzed by NIMS (Fig. 6). After ~8 h of hydrolysis at 60 °C, ~33 % of total hexose and ~56 % of total pentose sugars present in the biomass were solubilized by CelEcc alone. Four of the hybrids gave increased yield of total hexose products relative to CelEcc, with CelEcc_CBM44 giving an ~50 % yield for conversion of the cellulosic fraction of biomass to soluble products and ~60 % yield for conversion of the hemicellulose fraction of biomass to soluble products.

**Fig. 7:**
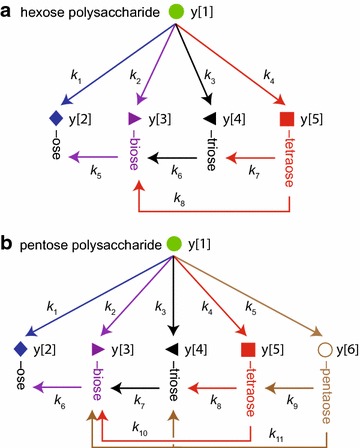
Kinetic schemes for enzymatic hydrolysis of cellulose and hemicellulose accounting for all products detected by quantitative NIMS analysis. **a** Cellulose hydrolysis leading to the release of soluble hexose sugars and subsequent conversions of the solubilized oligosaccharides. **b** Hemicellulose hydrolysis leading to soluble pentose sugars and subsequent conversions of the soluble oligosaccharides

**Fig. 8:**
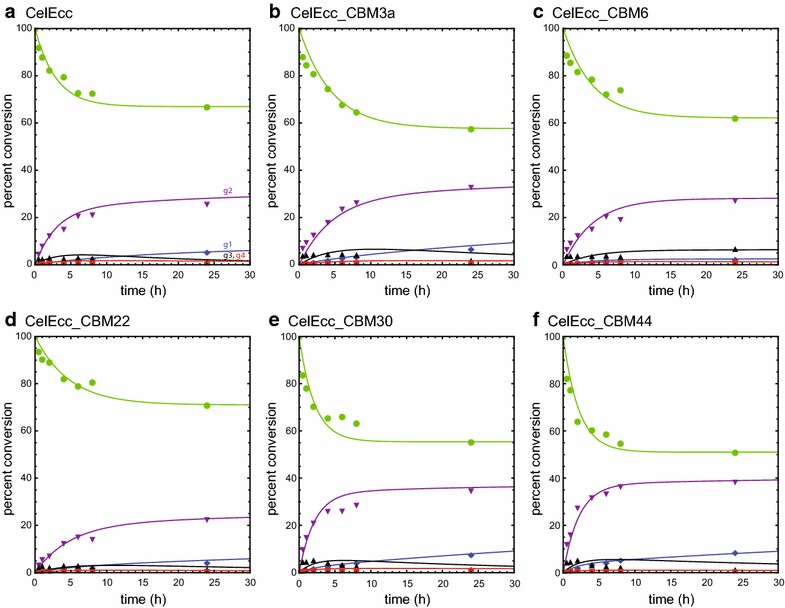
Analysis of the time course for formation of individual hexose products. CelEcc_CBMs used in this experiment were expressed in *E. coli* and purified as described in “Methods”. Reactions included CelEcc_CBM hybrids with IL-SG (0.32 µmol enzyme and 1 mg biomass in a total reaction volume of 0.1 mL). Cellulose fraction in unreacted biomass (*green* *solid circles*); glucose (g1, *blue diamonds*); cellobiose (g2, *purple down triangles*); cellotriose (g3, *black* *up triangles*); cellotetrose (p4, *red* *squares*). **a** CelEcc. **b** CelEcc_CBM3a. **c** CelEcc_CBM6. **d** CelEcc_CBM22. **e** CelEcc_CBM30. **f** CelEcc_CBM44. *Solid lines* are the results of simulation based on the kinetic scheme in Fig. 7a and the differential equations shown in Additional file 3: Differential equations

**Fig. 9:**
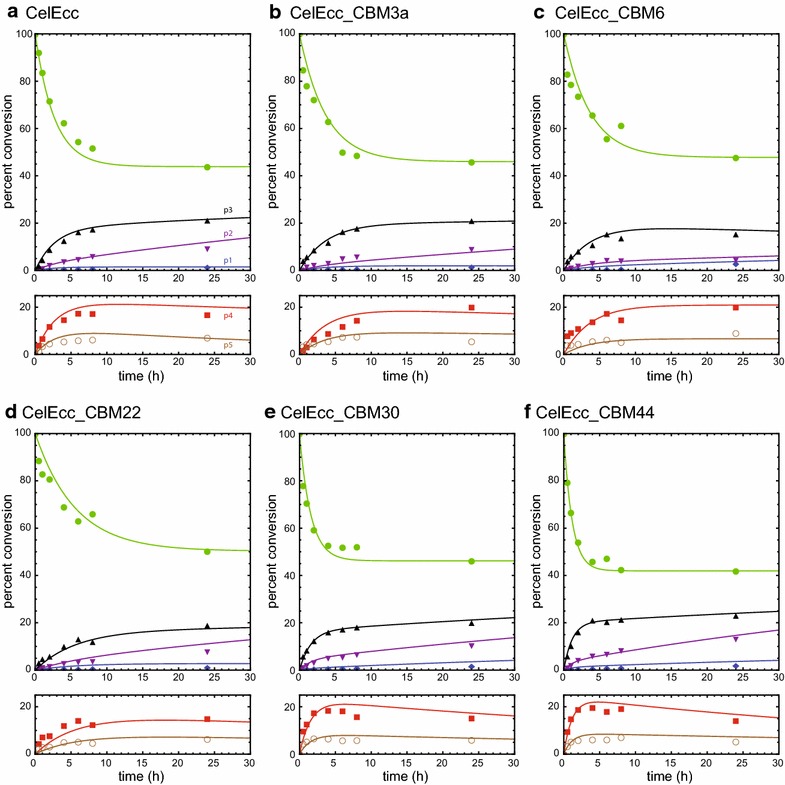
Analysis of the time course for formation of individual pentose products. CelEcc_CBMs used in this experiment were expressed in *E. coli* and purified as described in “Methods”. Reactions included CelEcc_CBM hybrids (0.32 µmol enzyme and 1 mg biomass in a total reaction volume of 0.1 mL) with IL-SG. Hemicellulose fraction in unreacted biomass (*green* *solid circles*); pentose (p1, *blue* *diamonds*); pentobiose (p2, *purple* *down triangles*); pentotriose (p3, *black* *up triangles*); pentotetraose (p4, *red* *squares*); pentopentaose (p5, *brown* *open circles*). **a** CelEcc. **b** CelEcc_CBM3a. **c** CelEcc_CBM6. **d** CelEcc_CBM22. **e** CelEcc_CBM30. **f** CelEcc_CBM44. *Solid color lines* are the results of simulation based on the kinetic scheme in Fig. 7b and the differential equations shown in Additional file 3: Differential equations

Figure 7 shows kinetic schemes that account for the products observed by quantitative NIMS from the reaction of CelEcc_CBM hybrids with IL-SG. These schemes assign apparent rate constants that account for release of soluble products from the insoluble biomass and subsequent conversion of soluble oligosaccharides into smaller molecules [61]. By use of NIMS, cascades of products from both the hexose and pentose fractions of the biomass can be monitored simultaneously, and the time courses for products observed are shown in Fig. 8 (hexose fraction) and Fig. 9 (pentose fraction).

Figure 8 shows the time course for the reaction of six CelEcc_CBM hybrids with the hexose fraction of IL-SG. The *solid* colored lines are results of simulations of the concentration of individual products based on the kinetic scheme of Fig. 7a and the differential equations shown in Additional file 3: Differential equations. Values for the apparent rates (see “Discussion”) determined from the numerical integration are presented in Table 4. As observed previously for CelEcc_CBM3a reactions [61], cellobiose (g2, *purple* down triangles) is the dominant product for reaction of each of the CelEcc_CBM fusions with the cellulosic fraction in IL-SG. Comparison of the progress curves for cellobiose formation shows that fusion of CelE to different CBMs changed both the magnitude of apparent rates and the overall yield for production of cellobiose. For example, CelEcc_CBM22 had the smallest apparent rates and overall yield, while CelEcc_CBM44 had the largest apparent rates and highest yield.

**Table 4:** Apparent rates obtained from numerical integration of NIMS time course data

CBM fusion^b^	None	CBM3a	CBM6	CBM22	CBM30	CBM44
Protein (nmol)^c^	0.32	0.32	0.32	0.32	0.32	0.32
Total hexose added (mM)^d^	26.1	26.1	26.1	26.1	26.1	26.1
Fraction hexose reacted^e^	0.33	0.42	0.38	0.29	0.45	0.49
Apparent rate^a^						
k1	0.013	0.011	0.009	0.009	0.017	0.027
k2	0.225	0.103	0.111	0.099	0.186	0.232
k3	0.049	0.024	0.025	0.024	0.035	0.028
k4	0.000	0.000	0.004	0.001	0.084	0.000
k5	0.044	0.016	0.007	0.002	0.011	0.003
k6	0.000	0.009	0.001	0.022	0.016	0.010
k7	0.000	0.003	0.000	0.008	0.000	0.000
k8	0.007	0.000	0.003	0.010	0.000	0.011
Total pentose added (mM)^d^	13.9	13.9	13.9	13.9	13.9	13.9
Fraction pentose reacted^e^	0.56	0.54	0.52	0.50	0.54	0.58
Apparent rate^a^						
k1	0.006	0.003	0.004	0.003	0.008	0.006
k2	0.028	0.016	0.016	0.009	0.031	0.045
k3	0.110	0.061	0.062	0.047	0.120	0.191
k4	0.002	0.063	0.074	0.041	0.096	0.331
k5	0.352	0.082	0.023	0.008	0.071	0.096
k6	0.002	0.002	0.000	0.000	0.000	0.000
k7	0.004	0.007	0.002	0.004	0.003	0.013
k8	0.000	0.004	0.006	0.000	0.000	0.001
k9	0.009	0.002	0.001	0.002	0.000	0.000
k10	0.001	0.001	0.000	0.000	0.003	0.007
k11	0.005	0.011	0.005	0.004	0.058	0.006

^a^Determined by numerical analysis according to reaction schemes shown in Fig. 7; differential equations used in the numerical analysis are shown in Additional file 3

^b^CelEcc fused to the indicated CBM

^c^Total nmol of CelEcc_CBM hybrid added to reaction

^d^Total hexose or pentose monomer added to the reaction as determined from exhaustive hydrolysis of IL-SG

^e^Fraction of total hexose or pentose present in the reaction converted to soluble products detected by quantitative NIMS analysis

Figure 9 shows the time course for the reaction of six CelEcc_CBM hybrids with the pentose fraction of IL-SG. *Solid* lines are derived from analysis of Fig. 7b as described above; the *dotted* black line represents the sum of the amounts of the individual products. With the pentose fraction, pentotriose (p3, *black* up triangles) is the dominant product for reaction of all of the CelEcc_CBM fusions, as observed earlier for CelEcc_CBM3a [61]. Although all of the CelEcc_CBM hybrids gave a similar yield of pentotriose at the endpoint of reaction (24 h), there were substantial differences in the magnitude of the dominant apparent rate (k3) associated with its formation (Table 4). Thus, CelEcc_CBM22, CelEcc_CBM6 and CelEcc_CBM3a were least effective at enhancing the rate for pentotriose accumulation, while CelEcc, CelEcc_CBM30 and CelEcc_CBM44 were most effective. The ability of CBM30 and CBM44 to promote both rapid hydrolysis and high yield of the pentose fraction can also be compared with the reaction of CelEcc (lacking a CBM), which did not promote rapid hydrolysis, but did achieve comparable yield after 24 h of reaction (Table 4; Fig. 6).

## Discussion

To begin this study, we created a plasmid that allows convenient fusion of two protein domains separated by a polypeptide linker sequence. Each of these individual parts can be iterated against each other by using four well-behaved restriction enzymes. Using this vector, a series of GFP_CBM expression plasmids were created. The fusion proteins were produced using cell-free translation, and the binding specificities of the GFP_CBMs were measured using soluble and insoluble pure polysaccharides and biomass (Tables 1, 2, 3; Figs. 3, 4, 5; Additional file 1: Tables S5, S6). Using the single broad specificity enzyme CelE as the catalytic domain, we were also able to examine the function of enzyme_CBM fusions against a range of substrates in a controlled manner (Figs. 4, 6).

All of the GFP_CBM and CelEcc_CBM constructs made were successfully expressed using cell-free translation. At least 100 µg of the GFP_CBMs and 30 µg of the individual CelEcc_CBMs were produced by 50 µL cell-free translation and used in described assays. Thirty-four of the GFP_CBMs produced in cell-free translation bound at least one substrate, and these results are overall consistent with previously determined binding specificities [40]. For example, CBM3a did not bind to any of the soluble substrates, but interacted strongly with insoluble cellulose and biomass in pull-down assays. Since CBM3a enhances activity towards insoluble cellulosic materials [41, 61, 66–68, 78, 79], it is not surprising that most of the CBMs studied herein were less active than CelEcc_CBM3a on PASC. Interestingly, an ~fourfold increase for CelEcc_CBM30 was determined for reaction with PASC relative to CelEcc_CBM3a. Enhanced reactivity of CelEcc_CBM30 can be rationalized by the demonstrated binding of CBM30 to both HEC and PASC [80] and increased reactivity of a GH9 cellulase with crystalline cellulose [67]. Although we observed CBM6 binding to lichenan, the enhancement of the CelE reaction with PASC (Fig. 4) could be due to the weak interaction of CBM6 with β-1,4-linked glucan reported by Czjzek et al. [81].

CBM22-2 produced in wheat germ extract was able to bind xylan, and this is consistent with the observation that CBM22s are primarily associated with xylanases and have been shown to bind xylan [82–84]. However, CelEcc_CBM22 was not particularly effective at hydrolyzing the pentose fraction in IL-SG (Table 4; Fig. 9). Likewise, GFP_CBM6 (from xylanase XynA, Cthe_2972) bound to beechwood xylan and arabinoxylan [81], but pairing CelE with this CBM gave only modest catalytic results with xylans. For both xylan and mannan, CelEcc_CBM44 showed more than a twofold enhancement in reactivity relative to CelEcc_CBM3a. CBM44 is part of CelJ (Cthe_0624), an enzyme that we showed had weak multifunctional behavior in reaction with IL-SG [61]. By combining CBM44 (diverse binding specificity) with CelE (multifunctional catalysis), we were able to create a fusion hybrid with improved reactivity.

It is also worth noting that some CBMs did not improved the catalytic activity of CelE, even though the CBM independently showed binding to one or more substrates. Thus, the lack of a correct orientation of the CBM relative to the CelE catalytic domain may influence binding and/or reactivity. For example, CBM16 and CBM54 are naturally found at the N-terminus of the catalytic domain (Additional file 1: Table S1) and perhaps need to be in this arrangement to enhance the reactivity of the catalytic domain. The linker between CelEcc and the CBM used in this study may also influence reactivity. In previous studies, linker lengths, compositions, orientations, and conformations were reported to have significant effects on enzyme reactivity [85–87]. The linker used in this study is naturally found in the CipA scaffoldin of the *R. thermocellum* cellulosome (amino acids 323–364 of Cthe_3077), and has some differences to other naturally occurring linker sequences. Half of ~40 residues of the linker used in this study are threonines and prolines, which possibly promote a flexible, extended conformation between domains [86–88]. Similarly, the linkers that connect CBM6 and CBM44 to the catalytic domain are ~25–30 residues long with multiple prolines and threonines that could be in an extended conformation In contrast, the linker in native CBM22-2 is ~25–30 residues long with only a few threonine residues, and the linker between CBM30 and the adjacent GH9 catalytic domain is less than 10 residues long, possibly indicating close association with the catalytic domain. CBMs are also found at the C-terminus relative the catalytic domain, the same as in our CelE_CBM constructs, while CBM22-2 and CBM30 are generally found at the N-terminus relative to the catalytic domain. Thus, it is possible that reactivity observed in the CBM22-2 and CBM30 constructs is influenced by an improper domain orientation.

We found that the highest binding affinity (*K*, Table 3) did not always predict the highest enzymatic activity. For example, CBM3a and CBM44 had the highest affinities for PASC, but did not give the highest reactivity with this substrate. In contrast, CBM30 showed weaker binding to PASC than other CBM constructs, but CelEcc_CBM30 showed the highest reactivity with PASC. For lichenan, all CBMs that had detectable binding affinities also increased the reactivity of CelE relative to CelEcc_CBM3a. However, although CBM44 had higher affinity for lichenan than CBM30, CelEcc_CBM30 showed ~2× faster reaction with lichenan than CelEcc_CBM44. Similarly, while GFP_CBM6 had a higher affinity for xylan than GFP_CBM44, CelEcc_CBM44 had the highest xylan reactivity. These trends support the previous conclusion that tight binding of a CBM (possibly reflecting dominance of *k*_on_ over *k*_off_ for interaction with the polysaccharide) may limit the number of productive hydrolytic events by the catalytic domain. If a CBM binds too tightly, the catalytic domain may not easily access new glycosidic bonds during the time duration when the CBM is adsorbed, thus restricting the diffusion of the catalytic domain [50, 52].

The binding-interaction constants provided by simulation using the logistic equation (Eq 6) also give insight into the interactions of the CBMs with the substrates. Most *c*-values, similar to Hill constants [89], shown in Table 3 are close to 1, indicating no higher order contributions to binding. However, the *c*-values >1 determined for CBM6 and CBM30 possibly indicate cooperative binding. Multiple members of the CBM6 family have been shown to have two binding sites with different binding specificities [81, 90], perhaps reflecting this possibility. In contrast, *c*-values <1 suggest noncooperative binding. This may be due to modifications to substrate/polysaccharide chain confirmations, as has been seen in starch-binding proteins [91], along with other possibilities such as steric occlusion of preferred binding sites and others.

The range of polysaccharides tested represents many of the most common plant carbohydrates found in ionic liquid treated biomass, including amorphous forms of cellulose and mixed-linkage β-glucan, and branched (arabinoxylan, oat spelt xylan, and galactomannan) and unbranched hemicellulose (beechwood and birchwood xylans; and 1,4-β-d-mannan). Among the CBMs tested, the majority were able to bind to linear, soluble hexose chains (e.g., HEC, lichenan, and PASC, Tables 1, 2), while fewer bound to Avicel and the hemicellulosic substrates, either linear or branched. However, several CBMs, including CBM6 and CBM44 bound to arabinoxylan (Table 1), which has partial branching [8]. These two CBMs also gave enhanced catalysis with the hemicellulosic fraction in IL-SG (Table 4; Fig. 9). Ionic liquid pretreatment of switchgrass, which has been used on the biomass used in this work, converts cellulose to an amorphous state and retains the hemicellulose [76, 77, 92]. The crystal structure of CelE shows a large, wide active site, which allows reactivity with multiple substrates (C. M. Bianchetti, T.E. Takasuka and B.G. Fox, unpublished data). This active site appears to be well structured to support reactions with amorphous forms of cellulose and hemicellulose, but is not as reactive with crystalline cellulose [61]. CBMs included in this work have capability for binding crystalline, linear, and branched polysaccharides [42, 80, 81], providing a useful diversity to match the properties of CelE.

Studies of time-dependent biomass hydrolysis using the quantitative NIMS assay and numerical simulation warrant a couple of closing comments. We describe the results of the numerical analysis as apparent rates for the appearance of soluble products, and in doing so acknowledge the complexity of the molecular-level events contributing to each of the steps given in the kinetic schemes of Fig. 7. These schemes may underestimate the total activity of the enzyme, for example, if a hydrolysis reaction does not yield a soluble product [93] detected by the NIMS analysis. With this caveat, an individual dominant apparent rate such as k2 for release of cellobiose from the hexose fraction or k3 for release of pentotriose from the pentose fraction will include contributions from a number of microscopic steps such as the accessibility of suitable sites on the substrate for catalysis (a property of the CelE active site) and binding affinity constants (*K*) for polysaccharide binding (influenced by the CBMs in this work). Other microscopic steps that can affect apparent rates include chemical steps in catalysis within the enzyme active site (which will be the same in this study), product release, the presence of alternative substrates and product inhibitors (possibly including soluble oligosaccharides and some branched polysaccharide products), changes in the composition of the remaining substrate, and perhaps others [61, 94, 95]. The systematic iteration of CBMs versus a single multifunctional catalytic domain offers a powerful tool to examine some of these critical aspects of the interactions of enzymes with biomass substrates and products.

## Conclusions

The results show that wheat germ cell-free translation can be productively used to screen the properties of CBMs as binding domains and as enhancers of catalytic activity. We have shown that fusions to different CBMs can alter the reactivity with four different polysaccharides. The combination of broad binding specificity and moderate binding affinity in the CBM with a single multifunctional GH5 catalytic domain gave best catalysis with plant biomass. We also showed that CelEcc_CBM44 alone was able to hydrolyze half of the cellulose and hemicellulose present in IL-SG in a short time regime (6 h). Other CelEcc_CBM hybrids achieved a similar endpoint yield of soluble products, albeit at slower rates. The approach of fusing different CBMs to multifunctional catalytic domains has potential to facilitate creation of new enzyme_CBM hybrids with improved reactivity for specific polysaccharide substructures within the complexity of plant biomass.

## Methods

### Cloning of GFP_CBM

Additional file 1: Table S1 summarizes properties of the genes from where the selected CBMs were extracted, their amino acid sequences, and molecular weights. Nucleotide sequences were retrieved from NCBI (http://www.ncbi.nlm.nih.gov/nuccore) and UniProt (http://www.uniprot.org/uniprot/ [96]). Nucleotide sequences encoding each CBM domain were selected, and PCR primer pairs were designed (Additional file 1: Table S2) to amplify the gene fragment of interest [60]. The forward primer of 5′-GCGAACACCCTTAAG-3′ was followed by the gene specific sequence encoding the N-terminal sequence of the CBM, while the reverse primer of 5′-TCTAGAGGATCCTTA-3′ was followed by the gene specific sequence encoding the C-terminal sequence of the CBM. The forward and reverse primers provided *Afl*II and *Bam*HI sites at the 5′- and 3′-ends of the amplicon, respectively. *R. thermocellum* ATCC 27405 genomic or synthetic (Additional file 1: Table S3) DNAs were used as PCR templates, and amplified PCR products were digested using *Afl*II and *Bam*HI (Promega, Madison WI). The different CBM sequences were ligated into the C-terminal domain position, which is flanked by *Afl*II and *Bam*HI restriction sites. The nucleotide sequence encoding the protein linker between the N- and C-terminal domains (protein sequence of *N*-NATPTKGATPTNTATPTKSATATPTRPSVPTNTPTNTPANT-*C*) was not modified from the parent CipA sequence. Plasmids isolated from successful transformations were sequence-verified by using the universal forward and reverse primers shown in Additional file 1: Table S2 at the University of Wisconsin-Madison Biotechnology Center.

### Cloning of CelEcc_CBM

GFP_CBM constructs produced as described above and a previously created CelEcc_CBM3a plasmid were separately digested with *Sgf*I and *Pme*I in order to obtain the nucleotide sequences encoding the CBM and CelEcc fragments. The digested plasmid and insert fragments were then gel-purified and ligated to form the CelEcc_CBM fusion plasmids [60]. All CelEcc_CBM plasmids were sequence-verified as described above.

### Cell-free translation

Plasmids encoding the individual GFP_CBM or CelEcc_CBM hybrids were prepared by mini prep (Qiagen, Germany) and cell-free protein syntheses [60, 61]. After mini prep, the plasmid DNA was treated with proteinase K (Sigma-Aldrich, St. Louis, MO, USA) in 10 mM Tris–HCl, pH 8.0, 5 mM EDTA and 0.1 % (w/v) SDS for 1 h at 37 °C to remove contaminating RNase. The proteinase K treatment was followed by phenol/chloroform extraction and ethanol precipitation. After ethanol precipitation, the concentration of plasmid DNA was measured using a Nanodrop 2000C spectrometer (Thermo Fisher Scientific, Waltham, MA, USA). The plasmid DNA was adjusted to 1 µg/µL for use in the transcription and translation reactions. A Protemist DT-11 robot (CellFree Sciences, Yokohama, Japan) carried out the transcription and translation reactions using plasmid DNA, premade transcription and translation mixtures, and translation buffer.

For transcription, 5 µL of the plasmid DNA was added to 45 µL of transcription mixture (80 mM HEPES–KOH, pH 7.8, 20 mM magnesium acetate, 2 mM spermidine hydrochloride, 10 mM DTT, 2.5 mM NTPs, 50 units of SP6 RNA polymerase, and 50 units of RNase inhibitor (CellFree Sciences, Yokohama, Japan) and then incubated for 4 h at 37 °C to produce transcripts.

The wheat germ cell-free translation reaction was performed by bilayer method for 24 h at 37 °C with 59 µL of translation mixture [containing 56 µL of WEPRO2240H wheat germ extract (CellFree Sciences, Yokohama, Japan), 0.1 mM amino acids mix, and 0.07 µg/µL creatine kinase (Roche, Basel, Switzerland)] and 1.1 mL of translation buffer [1× Solutions 1, 2, 3 and 4 (CellFree Sciences, Yokohama, Japan)]. Translated proteins were visualized by SDS-PAGE, and the amount of produced protein was estimated using a gel imager (Bio-Rad, Hercules, CA, USA) (Additional file 1: Table S4). Cell-free reactions with empty vector were used as controls for protein translation, and in enzyme and pull-down assays.

Translated GFP_CBMs were used in pull-down assays with insoluble polysaccharides without further purification from the translation reaction mixture. Translated GFP_CBMs used in affinity gel electrophoresis were purified from the translation reaction mixture using Ni beads (GE Healthcare, Piscataway, NJ, USA) [60]. The translated protein was incubated and mixed with Ni beads in 100 mM MOPS, pH 7.4, containing 300 mM NaCl, 2 mM CaCl_2_ and 25 mM imidazole to bind protein to beads. Then, the Ni beads were washed three times with 100 mM MOPS, pH 7.4, containing 300 mM NaCl, 2 mM CaCl_2_ and 50 mM imidazole. Protein was eluted twice and combined from the Ni beads with 100 mM MOPS, pH 7.4, containing 300 mM NaCl, 2 mM CaCl_2_, and 250 mM imidazole. The purified protein was buffered exchanged into 100 mM MOPS, pH 7.4, containing 50 mM NaCl, and 2 mM CaCl_2_ using VIVASPIN500 (Sartorius Stedim, Bohemia, NY, USA).

CelEcc_CBMs prepared using cell-free translation were used in enzyme assays without further purification from the translation reaction mixture. Previous studies have established that the wheat germ extract has no endogenous enzymes capable of reacting with polysaccharides studied here [60]. All proteins synthesized by cell-free translation were checked for fractional solubility by SDS-PAGE after centrifugation at 13,200×*g* for 10 min at 4 °C. Solubility was assessed by the ratio of intensities for the expressed protein remaining in the supernatant after centrifugation as compared to before centrifugation. All of the constructs described here showed greater than 95 % solubility after cell-free translation.

### Cloning, expression in *Escherichia coli*, and purification

Polymerase incomplete primer extension [97] was used to transfer the nucleotide sequences encoding the GFP_CBMs and CelEcc_CBMs from their respective pEUTTJW plasmids into the *E. coli* expression vector pVP67K [98]. The primer pair used to amplify the GFP_CBM and CelEcc_CBM genes (Additional file 1: Table S2) matches a portion of the sequence from pVP67K [98], while the primer pair used to amplify pVP67K matches the corresponding sequences on pVP67K. The PCR amplification of pEUTTJW and pVP67K were carried out in separate reactions. After the PCR, aliquots (2 µL) from the two PCR were mixed and immediately transformed into competent *E. coli* BL21-CodonPlus (DE3)-RILP cells (Agilent Technologies, Santa Clara, CA, USA). The transformed cells were plated onto LB agar plates containing 50 µg/mL kanamycin and 34 µg/mL chloramphenicol, and viable transformants were screened for plasmids containing inserts. GFP_CBM and CelEcc_CBM inserts in pVP67K were sequence-verified as described above using the universal forward and reverse primers shown in Additional file 1: Table S2. Further details on the construction and reactivity of CelEcc and CelEcc_CBM3a are provided in a previous study [61].

*E. coli* BL21-CodonPlus (DE3)-RILP cells containing a GFP_CBM, CelEcc or CelEcc_CBM expression plasmid were grown in 10 mL of noninducing medium [98] containing 50 µg/mL kanamycin and 34 µg/mL chloramphenicol for 12 h at room temperature, and then transferred into 500 mL of auto-induction medium containing the same antibiotics for 16 h at 37 °C [98]. Cells were harvested by centrifugation at 5000×*g* for 20 min. The cell pellet was suspended in 20 mM Tris HCl, pH 7.0, containing 1 mM EDTA, a protease inhibitor cocktail containing 1 µM E-64 (Sigma-Aldrich, St. Louis, MO, USA), and 0.5 mM benzamidine (Calbiochem, Spring Valley, CA, USA). The suspended cells were sonicated with a cycle of 15 s on and 15 s off for 10 min on ice. The sonicated cells were centrifuged at 20,000 rpm for 60 min at 4 °C, and the supernatant was loaded onto a HisTrap column (1.6 cm dia × 2.5 cm bed height, GE Healthcare, Piscataway, NJ, USA) equilibrated with 100 mM MOPS, pH 7.0, containing 500 mM NaCl. After loading, the column was washed with 10 volumes of the same buffer. The bound protein was eluted with a linear 100 mL gradient of 100 mM MOPS, pH 7.0, containing 500 mM NaCl and 0.5 M imidazole. To cleave the His-tag from the N-terminus of the fusion protein, 40 µg of His-tagged tobacco etch virus (TEV) protease was mixed with 1 mg of the protein sample, and incubated for 12 h at 4 °C [99]. Subtractive immobilized metal affinity chromatography was used to separate the His-tag-free protein from unreacted sample and His-tagged TEV protease. The His-tag-free protein was concentrated using VIVASPIN20 (Sartorius Stedim, Bohemia, NY, USA) at 4200×*g* to a final concentration of ~10 mg/mL. The His-tag was not cleaved from the N-terminus of GFP_CBM constructs. The protein concentration was estimated by BCA assay (Bio-Rad, Hercules, CA, USA), as well as spectrophotometrically at 280 nm by using the extinction coefficients calculated from the amino acid sequences of the constructs.

### Substrates

Hydroxyethyl cellulose (HEC) and beechwood xylan (≥90 % xylose) (Sigma-Aldrich, St. Louis, MO, USA), icelandic moss lichenan (84 % glucose), carob galactomannan (22 % galactose, 78 % mannose) and wheat flour arabinoxylan (51 % xylose, 36 % arabinose) (Megazyme, Wicklow, Ireland) were used in affinity gel electrophoresis assays. Icelandic moss lichenan (Megazyme, Wicklow, Ireland), birchwood xylan (≥90 % xylose) and Avicel PH-101 (Sigma-Aldrich, St. Louis, MO, USA), phosphoric acid-swollen cellulose (PASC, prepared as described previously [100]), 1,4-β-d-mannan (98 % mannose) (Megazyme, Wicklow, Ireland), icelandic moss lichenan, ammonia fiber expansion pretreated switchgrass (AFEX-SG, [77, 101]) and ionic liquid pretreated switchgrass (IL-SG, [92]) were used for pull-down binding assays. AFEX-SG was used in binding studies without further handling, while IL-SG (400 mg) was used in binding studies and enzyme assays after being washed three times with 40 mL of autoclaved MilliQ water and then re-suspended in 20 mL of autoclaved MilliQ water. Icelandic moss lichenan, birchwood xylan, PASC, and 1,4-β-d-mannan, and washed IL-SG were used for enzyme assays. Oat spelt xylan (≥70 % xylose, ≤10 % arabinose, ≤15 % glucose) (Sigma-Aldrich, St. Louis, MO, USA), icelandic moss lichenan, PASC and 1,4-β-d-mannan were used for the binding affinity measurements. Oat spelt xylan used for the binding affinity assay was prepared [102, 103] by boiling two grams of the polysaccharide in 100 mL of distilled water for 30 min and subsequently pelleting the insoluble fraction by centrifugation for 20 min at 4300×*g* at 4 °C. The insoluble xylan pellet was washed three times by centrifugation for 20 min at 4300×*g* at 4 °C and placed at −80 °C overnight. The sample was lyophilized to obtain ~1 g of the final insoluble substrate. The composition of IL-SG was determined using NREL procedures LAP-002 and LAP-005 [92, 104, 105]. The IL pretreatment causes removal of lignin and some hemicellulose, thereby enriching the fraction of glucan. The fractional sugar content of IL-SG used as the enzyme substrate (1 mg) was glucose (0.47 mg); xylose (0.18 mg), arabinose (0.03 mg), other sugars, lignin, and ash (0.32 mg).

### Soluble substrate binding assay

Affinity gel electrophoresis was performed to test binding specificities of the GFP_CBMs to various soluble polysaccharides (HEC, icelandic moss lichenan, carob galactomannan, beechwood xylan, and wheat flour arabinoxylan) [40, 106]. Continuous 6 % polyacrylamide gels (29:1, acrylamide:bisacrylamide) containing 0.1 % (w/v) of soluble polysaccharide were prepared with Bio-Rad Criterion empty cassettes and 26-well combs (Bio-Rad, Hercules, CA, USA) in the presence of 2 mM CaCl_2_. Soybean trypsin inhibitor (STI, 5 μg) (Sigma-Aldrich, St. Louis, MO, USA) was used as an internal loading standard, and approximately 150 ng of each purified GFP_CBM was used for affinity gel electrophoresis. Electrophoresis was performed at 4 °C and pH 8.3 for 75 min at a constant voltage of 150 V in a Criterion Electrophoresis cell (Bio-Rad, Hercules, CA, USA). Gels were silver-stained to detect the protein [107]. Briefly, the gels were soaked in fixing solution (500 mL methanol, 120 mL acetic acid, 0.5 mL 37 % formaldehyde in a total volume of 1 L made up with deionized water) for 1 h, washed three times in 50 % ethanol for 5 min, and then treated with 0.81 mM Na_2_S_2_O_3_∙5H_2_O for 1 min. The gels were rinsed three times with de-ionized water for 20 s and then placed in staining solution (12 mM AgNO_3_, 0.75 mL/L 37 % formaldehyde) for 1 h. The gels were rinsed an additional three times with de-ionized water for 20 s, then placed in developing solution (0.57 M Na_2_CO_3_, 0.5 mL/L 37 % formaldehyde, 20 μM Na_2_S_2_O_3_∙5H_2_O) for 5 to 10 min, and rinsed two times with de-ionized water for 5 s. The development was halted with 50 % methanol, 12 % acetic acid for 10 min, and washed in 50 % methanol for 20 min. Gel images were obtained using the Gel Doc EZ system (BioRad), and analyzed for the presence of binding by the calculation of relative mobility ratios (*R*_r_) and visual inspection. *R*_r_ values were calculated by the following equation:

1 $$\begin{aligned}R_{\text{r}} &= \frac{{R_{\text{p}} }}{{R_{\text{n}} }} \hfill \\ &= \frac{{\left[ {{\text{GFP}}\_{\text{CBM migration (mm)}}/{\text{STI migration (mm)}}} \right]\;{\text{with substrate}}}}{{\left[ {{\text{GFP}}\_{\text{CBM migration }}({\text{mm}})/{\text{STI migration (mm)}}} \right]\;{\text{without substrate}}}} \end{aligned}$$

where *R*_p_ is the relative mobility of a GFP_CBM compared to STI in the presence of substrate, and *R*_n_ is the relative mobility of a GFP_CBM compared to STI in the absence of substrate [40, 106]. A *R*_r_ less than 0.750 was chosen to indicate GFP_CBM binding to decrease the chances of observing false-positive binding. The *R*_r_ values are listed in Additional file 1: Table S5.

### Insoluble substrate pull-down assay

Pull-down assays were used to test binding specificities of the GFP_CBMs to insoluble polysaccharides (Avicel PH-101, PASC, 1,4-β-d-mannan, birchwood xylan, AFEX-SG, and IL-SG). Aliquots (25 µL) of cell-free expressed, unpurified GFP_CBM were mixed with 1 mg of substrate in a final volume of 100 μL in 96-well microtiter plates, giving a final reaction concentration of 10 mg/mL insoluble substrate in 50 mM MES, pH 6.0, containing 2 mM CaCl_2_. Pull-down assays of protein in the absence of substrate were performed as a control, and all binding experiments were done in triplicate. Samples were incubated for 1 h at 4 °C and shaken at 600 rpm with a Thermo Scientific Titer Plate Shaker (Model No. 4625) (Thermo Fisher Scientific, Waltham, MA, USA), and then spun at 4300×*g* for 10 min at 4 °C. Aliquots (20 µL) of the sample supernatants were mixed with 20 μL of de-ionized water, and the fluorescence was measured with excitation at 488 nm and excitation at 510 nm. Supernatant aliquots of the no-substrate samples were taken before the 10-min spin for use as the total fluorescence control to account for any protein precipitation during subsequent calculations. Cell-free expressed GFP alone was also assayed to determine if there were interactions between GFP and the insoluble substrates tested. To calculate the substrate-bound fraction of a GFP_CBM, pellet fluorescence was calculated for each substrate/no substrate combination (*F*_s_ and *F*_ns_) by subtracting the supernatant fluorescence, *f*, from total fluorescence, *T*, in the no-substrate reaction sample before the 10-min spin.

2 $$F = T - f$$

Normalized pellet fraction percentages (PF %) were calculated to account for protein precipitation. The no-substrate pellet fluorescence, *F*_ns_, was subtracted from the pellet fluorescence of a substrate-containing reaction, *F*_s_, and then divided by *T* and multiplied by 100.

3 $${\text{PF\,\% }} = \frac{{(F_{\text{s}} - F_{\text{ns}} )}}{T} \times 100$$

The PF % determined from GFP alone was subtracted from the PF % of the GFP_CBM constructs to remove the influence of GFP-substrate interactions in the observed PF %. A normalized PF % (with GFP PF % subtracted) of 10 % or greater was chosen to indicate GFP_CBM binding to decrease the chances of observing false-positive binding. The normalized PF % values are listed in Additional file 1: Table S6.

### Insoluble substrate binding affinity measurements

A range from 0 to 10 mg/mL of PASC, icelandic moss lichenan, 1,4-β-d-mannan, and oat spelt xylan were mixed with 0.5 μM of a GFP_CBM in a final volume of 400 μL of 25 mM Tricine, pH 8.0, with 188 mM NaCl, 2 mM CaCl_2_, and 1 mg/mL BSA in 2.0-mL microcentrifuge tubes. Binding reactions were carried out in triplicate. Control reactions with GFP_CBM at concentrations ranging from 0 to 0.85 μM in the absence of substrate were used to create standard curves to determine the amount of unbound protein remaining in the supernatant of a reaction containing substrate. Reactions were incubated for 1 h at 4 °C and shaken at 1200 rpm using an Eppendorf Thermomixer R (Eppendorf North America, Hauppauge, NY, USA) followed by centrifugation at 4300×*g* for 10 min at 4 °C. The fluorescence of 200 μL aliquots of reaction supernatants was measured with excitation at 488 nm and excitation at 510 nm. *E. coli*-expressed GFP was used as a control for nonspecific interactions of the GFP domain.

The fraction of GFP_CBM bound, *θ*, was calculated using Eqs. 4 and 5

4 $$B = 0.5 \;\mu {\text{M}} - \frac{f}{m}$$

5 $$\theta = \frac{B}{{0.5 \mu {\text{M}}}}$$

where *B* represents bound concentration, 0.5 μM representing the concentration of GFP_CBM added to the reaction, *f* representing supernatant fluorescence, and m representing the slope of the standard curve for no-substrate sample supernatant fluorescence versus GFP_CBM concentration. GFP_CBM fraction bound values were plotted versus substrate concentrations (mg/mL), [*S*], and the plots were used to determine dissociation constants (mg/mL), *K*, and binding-interaction constants, *c*, using a logistic equation as the binding model and fitting

6 $$\theta = \frac{{[S]^{c} }}{{K + [S]^{c} }}$$

Eq. (6). Dissociation constants were calculated using the NonlinearModelFit routine in Mathematica (Wolfram, Champaign, IL, USA).

### Enzyme assays with pure substrates

For reaction with PASC, a 15-μL aliquot of the cell-free translation reaction was combined with 35 μL of MES buffer, CaCl_2_ and PASC to give concentrations of 50 mM MES, pH 6.0, 2 mM CaCl_2_ and 10 mg/mL of PASC. This solution was reacted for 20 h at 60 °C. For reaction with either icelandic moss lichenan, birchwood xylan, or 1,4-β-d-mannan, a 5-μL aliquot of the translation reaction was combined with 45 μL of MES buffer, CaCl_2_, and substrate to give final concentrations of 50 mM MES buffer, pH 6.0, 2 mM CaCl_2_ and 10 mg/mL of polysaccharide and reacted for 20 h at 60 °C. DNS assays of reducing sugars were performed as described previously [108]. Briefly, 30 μL of supernatant from the reaction was mixed with 60 µL of DNS reagent and incubated for 5 min at 95 °C. The color change was monitored at 540 nm, and total reducing sugar content was estimated by comparison to standard curves prepared using d-glucose. All enzyme reactions were performed in triplicate.

### NIMS analysis of reactions with IL-SG

Synthesis of the *O*-alkyloxyamine fluorous-tagged NIMS reagent has been published [61]. Reactions of CelEcc and CelEcc_CBM variants were carried out in 50 mM phosphate, pH 6.0 and IL-SG present at 10 mg/mL. For these studies, the enzymes were expressed in *E. coli* and purified as described above. The concentrations of purified enzyme stock solutions were CelEcc (18 mg/mL, 38,230 Da); CelEcc_CBM3a (8 mg/mL, 60,118 Da); CelEcc_CBM6 (20 mg/mL, 55,460 Da); CelEcc_CBM30 (26 mg/mL, 64,687 Da); and CelEcc_CBM44 (5 mg/mL, 59,308 Da). Reactions were designed to contain equimolar amounts of enzyme-active sites (0.32 µmol), and all reactions were carried out at 60 °C for 24 h. At 1, 2, 4, 8, and 24 h, a 2-µL aliquot of the reaction mixture was transferred into a vial containing 6 µL of 100 mM glycine acetate, pH 1.2: 1.0 µL of a 2.5-mM aqueous solution of [*U*]-^13^C glucose; 2.5 mM aqueous solution of [*U*]-^13^C xylose; 2 µL of CH_3_CN; 2 µL of MeOH; 1 µL of the NIMS probe [100 mM in 1:1 (v/v) H_2_O:MeOH]; and 0.1 µL of aniline. Quenched reaction mixtures were incubated at room temperature for 16 h, and then a 0.12-µL aliquot was spotted onto the surface of the NIMS chip and removed after 30 s. A grid drawn manually on the NIMS chip using a diamond-tip scribe helped in spotting and identification of sample spots in the spectrometer. NIMS chips were loaded using a modified standard MALDI plate and analyzed using a 4800 MALDI TOF/TOF mass spectrometer (Applied Biosystems, Foster City, CA, USA). Signal intensities were identified for the ions of the tagging products, and ~1000 laser shots were collected for each sample spot. Product quantitation was achieved by means of either [*U*]-^13^C glucose or [*U*]-^13^C xylose as an internal standard.

For reactions of purified enzymes with biomass, the time dependence of product formation detected by NIMS was analyzed by nonlinear global optimization of differential equations accounting for the appearance and decay of products [61] using Mathematica routine NDSolve and the Nelder–Mead simplex method for constrained minimization [109]. The differential equations are shown in Additional file 3: Differential equations account for release of soluble oligosaccharides from biomass and their subsequent hydrolysis reactions to end products. Successive rounds of parameter optimization with adjustment of constraints were carried out until the sum of the squares difference between the calculated and the experimental values reached a minimum, and no parameter was artificially constrained.

## Additional files

10.1186/s13068-015-0402-0  Differential equations corresponding to the kinetic schemes shown in Fig. 7a (hexose products derived from cellulose) and in Fig. 7b (pentose products derived from hemicellulose).
10.1186/s13068-015-0402-0 Affinity gel electrophoresis of GFP_CBM fusions. Thirty-nine GFP_CBM fusions were tested for binding specificities in native polyacrylamide gels containing CaCl_2_ and either lichenan, galactomannan, beechwood xylan, or arabinoxylan. A “No substrate” gel is shown for comparison with the substrate gels. Red stars indicate where binding was detected. Soybean trypsin inhibitor (STI) was used as a control for no binding.
10.1186/s13068-015-0402-0 Differential equations corresponding to the kinetic schemes of Figure 7 are presented in Mathematica format.

## Abbreviations

GH: glycoside hydrolase
CBM: carbohydrate-binding module
CelEcc: broad specificity GH family 5 (GH5) domain from *R. thermocellum* Cthe_0797
IL-SG: ionic liquid pretreated switchgrass
AFEX-SG: ammonia fiber expansion pretreated switchgrass
LPMO: lytic polysaccharide monooxygenase
HEC: hydroxyethylcellulose
NIMS: nanostructure-initiator mass spectrometry
PASC: phosphoric acid-swollen cellulose

## References

[1] Lynd LR, et al. Microbial cellulose utilization: fundamentals and biotechnology. Microbiol Mol Biol Rev. 2002;66(3):506–77 **(table of contents)**. 10.1128/MMBR.66.3.506-577.2002 PMC120791 12209002

[2] Himmel ME (2007). Biomass recalcitrance: engineering plants and enzymes for biofuels production. Science.

[3] Furtado A (2014). Modifying plants for biofuel and biomaterial production. Plant Biotechnol J.

[4] Brett CT (2000). Cellulose microfibrils in plants: biosynthesis, deposition, and integration into the cell wall. Int Rev Cytol.

[5] Mosier N (2005). Features of promising technologies for pretreatment of lignocellulosic biomass. Bioresour Technol.

[6] Chundawat SP (2011). Deconstruction of lignocellulosic biomass to fuels and chemicals. Annu Rev Chem Biomol Eng.

[7] Scheller HV, Ulvskov P (2010). Hemicelluloses. Annu Rev Plant Biol.

[8] Pauly M (2013). Hemicellulose biosynthesis. Planta.

[9] Vanholme R (2010). Lignin biosynthesis and structure. Plant Physiol.

[10] Boerjan W, Ralph J, Baucher M (2003). Lignin biosynthesis. Annu Rev Plant Biol.

[11] Atalla RH, Vanderhart DL (1984). Native cellulose: a composite of two distinct crystalline forms. Science.

[12] Atalla RH, Vanderhart DL (1999). The role of solid state 13C NMR spectroscopy in studies of the nature of native celluloses. Solid State Nucl Magn Reson.

[13] Langan P, Nishiyama Y, Chanzy H (2001). X-ray structure of mercerized cellulose II at 1 a resolution. Biomacromolecules.

[14] Nishiyama Y, Langan P, Chanzy H (2002). Crystal structure and hydrogen-bonding system in cellulose Ibeta from synchrotron X-ray and neutron fiber diffraction. J Am Chem Soc.

[15] Nishiyama Y (2003). Crystal structure and hydrogen bonding system in cellulose I (alpha) from synchrotron X-ray and neutron fiber diffraction. J Am Chem Soc.

[16] Gilbert HJ, Stalbrand H, Brumer H (2008). How the walls come crumbling down: recent structural biochemistry of plant polysaccharide degradation. Curr Opin Plant Biol.

[17] Burton RA, Gidley MJ, Fincher GB (2010). Heterogeneity in the chemistry, structure and function of plant cell walls. Nat Chem Biol.

[18] Gibson LJ (2012). The hierarchical structure and mechanics of plant materials. J R Soc Interface.

[19] Meng X, Ragauskas AJ (2014). Recent advances in understanding the role of cellulose accessibility in enzymatic hydrolysis of lignocellulosic substrates. Curr Opin Biotechnol.

[20] Lynd LR (2008). How biotech can transform biofuels. Nat Biotechnol.

[21] Jordan DB (2012). Plant cell walls to ethanol. Biochem J.

[22] Kumar R, Singh S, Singh OV (2008). Bioconversion of lignocellulosic biomass: biochemical and molecular perspectives. J Ind Microbiol Biotechnol.

[23] Dodd D, Cann IK (2009). Enzymatic deconstruction of xylan for biofuel production. Glob Change Biol Bioenergy.

[24] Beguin P (1992). Bacterial cellulases. Biochem Soc Trans.

[25] Zhang YH, Himmel ME, Mielenz JR (2006). Outlook for cellulase improvement: screening and selection strategies. Biotechnol Adv.

[26] Shallom D, Shoham Y (2003). Microbial hemicellulases. Curr Opin Microbiol.

[27] Girio FM (2010). Hemicelluloses for fuel ethanol: a review. Bioresour Technol.

[28] Levasseur A (2013). Expansion of the enzymatic repertoire of the CAZy database to integrate auxiliary redox enzymes. Biotechnol Biofuels.

[29] Vaaje-Kolstad G (2010). An oxidative enzyme boosting the enzymatic conversion of recalcitrant polysaccharides. Science.

[30] Langston JA (2011). Oxidoreductive cellulose depolymerization by the enzymes cellobiose dehydrogenase and glycoside hydrolase 61. Appl Environ Microbiol.

[31] Hemsworth GR, Davies GJ, Walton PH (2013). Recent insights into copper-containing lytic polysaccharide mono-oxygenases. Curr Opin Struct Biol.

[32] Morgenstern I, Powlowski J, Tsang A (2014). Fungal cellulose degradation by oxidative enzymes: from dysfunctional GH61 family to powerful lytic polysaccharide monooxygenase family. Brief Funct Genomics.

[33] Kim IJ (2014). Synergistic proteins for the enhanced enzymatic hydrolysis of cellulose by cellulase. Appl Microbiol Biotechnol.

[34] Arfi Y (2014). Integration of bacterial lytic polysaccharide monooxygenases into designer cellulosomes promotes enhanced cellulose degradation. Proc Natl Acad Sci USA.

[35] Isaksen T (2014). A C4-oxidizing lytic polysaccharide monooxygenase cleaving both cellulose and cello-oligosaccharides. J Biol Chem.

[36] Hemsworth GR (2014). Discovery and characterization of a new family of lytic polysaccharide monooxygenases. Nat Chem Biol.

[37] Boraston AB (2004). Carbohydrate-binding modules: fine-tuning polysaccharide recognition. Biochem J.

[38] Cantarel BL, et al. The carbohydrate-active enzymes database (CAZy): an expert resource for glycogenomics. Nucleic Acids Res. 2009;37(Database issue):D233–8. 10.1093/nar/gkn663 PMC2686590 18838391

[39] Guillen D, Sanchez S, Rodriguez-Sanoja R (2010). Carbohydrate-binding domains: multiplicity of biological roles. Appl Microbiol Biotechnol.

[40] Abbott DW, Boraston AB (2012). Quantitative approaches to the analysis of carbohydrate-binding module function. Methods Enzymol.

[41] Morag E (1995). Expression, purification, and characterization of the cellulose-binding domain of the scaffoldin subunit from the cellulosome of *Clostridium thermocellum*. Appl Environ Microbiol.

[42] Tormo J (1996). Crystal structure of a bacterial family-III cellulose-binding domain: a general mechanism for attachment to cellulose. EMBO J.

[43] Abou Hachem M (2000). Carbohydrate-binding modules from a thermostable *Rhodothermus marinus* xylanase: cloning, expression and binding studies. Biochem J.

[44] Boraston AB (2002). Co-operative binding of triplicate carbohydrate-binding modules from a thermophilic xylanase. Mol Microbiol.

[45] Bolam DN (2004). X4 modules represent a new family of carbohydrate-binding modules that display novel properties. J Biol Chem.

[46] Abbott DW, Eirin-Lopez JM, Boraston AB (2008). Insight into ligand diversity and novel biological roles for family 32 carbohydrate-binding modules. Mol Biol Evol.

[47] Hall J (1995). The non-catalytic cellulose-binding domain of a novel cellulase from *Pseudomonas fluorescens* subsp. *cellulosa* is important for the efficient hydrolysis of Avicel. Biochem J.

[48] Jamal-Talabani S (2004). Ab initio structure determination and functional characterization of CBM36; a new family of calcium-dependent carbohydrate binding modules. Structure.

[49] Montanier C (2009). Evidence that family 35 carbohydrate binding modules display conserved specificity but divergent function. Proc Natl Acad Sci USA.

[50] Herve C (2010). Carbohydrate-binding modules promote the enzymatic deconstruction of intact plant cell walls by targeting and proximity effects. Proc Natl Acad Sci USA.

[51] Cuskin F (2012). How nature can exploit nonspecific catalytic and carbohydrate binding modules to create enzymatic specificity. Proc Natl Acad Sci USA.

[52] Gao D (2013). Increased enzyme binding to substrate is not necessary for more efficient cellulose hydrolysis. Proc Natl Acad Sci USA.

[53] Lamed R, Setter E, Bayer EA (1983). Characterization of a cellulose-binding, cellulase-containing complex in *Clostridium thermocellum*. J Bacteriol.

[54] Bayer EA (1998). Cellulose, cellulases and cellulosomes. Curr Opin Struct Biol.

[55] Doi RH, Kosugi A (2004). Cellulosomes: plant-cell-wall-degrading enzyme complexes. Nat Rev Microbiol.

[56] Fontes CM, Gilbert HJ (2010). Cellulosomes: highly efficient nanomachines designed to deconstruct plant cell wall complex carbohydrates. Annu Rev Biochem.

[57] Yutin N, Galperin MY (2013). A genomic update on clostridial phylogeny: Gram-negative spore formers and other misplaced clostridia. Environ Microbiol.

[58] Gnansounou E, Dauriat A (2010). Techno-economic analysis of lignocellulosic ethanol: a review. Bioresour Technol.

[59] Martinez D (2008). Genome sequencing and analysis of the biomass-degrading fungus *Trichoderma reesei* (syn. *Hypocrea jecorina*). Nat Biotechnol.

[60] Takasuka TE (2014). Cell-free translation of biofuel enzymes. Methods Mol Biol.

[61] Deng K (2014). Rapid kinetic characterization of glycosyl hydrolases based on oxime derivatization and nanostructure-initiator mass spectrometry (NIMS). ACS Chem Biol.

[62] Poole DM (1991). Characterization of hybrid proteins consisting of the catalytic domains of *Clostridium* and *Ruminococcus endoglucanases*, fused to *Pseudomonas* non-catalytic cellulose-binding domains. Biochem J.

[63] Fujimoto Z (2013). The structure of a *Streptomyces avermitilis* alpha-l-rhamnosidase reveals a novel carbohydrate-binding module CBM67 within the six-domain arrangement. J Biol Chem.

[64] Kim TW (2010). Binding modules alter the activity of chimeric cellulases: effects of biomass pretreatment and enzyme source. Biotechnol Bioeng.

[65] Liu W (2010). Engineering of *Clostridium phytofermentans* endoglucanase Cel5A for improved thermostability. Appl Environ Microbiol.

[66] Ye X (2011). Fusion of a family 9 cellulose-binding module improves catalytic potential of *Clostridium thermocellum* cellodextrin phosphorylase on insoluble cellulose. Appl Microbiol Biotechnol.

[67] Telke AA (2012). Construction and characterization of chimeric cellulases with enhanced catalytic activity towards insoluble cellulosic substrates. Bioresour Technol.

[68] Gao S (2014). New insights into enzymatic hydrolysis of heterogeneous cellulose by using carbohydrate-binding module 3 containing GFP and carbohydrate-binding module 17 containing CFP. Biotechnol Biofuels.

[69] Cubitt AB (1995). Understanding, improving and using green fluorescent proteins. Trends Biochem Sci.

[70] Tsien RY (1998). The green fluorescent protein. Annu Rev Biochem.

[71] Tsien RY (2009). Constructing and exploiting the fluorescent protein paintbox (Nobel Lecture). Angew Chem Int Ed Engl.

[72] Ding, SY, et al. Versatile derivatives of carbohydrate-binding modules for imaging of complex carbohydrates approaching the molecular level of resolution. Biotechniques. 2006;41(4):435–6 **(438, 440 passim)**. 10.2144/000112244 17068959

[73] Hong J, Ye X, Zhang YH (2007). Quantitative determination of cellulose accessibility to cellulase based on adsorption of a nonhydrolytic fusion protein containing CBM and GFP with its applications. Langmuir.

[74] Lim S, Chundawat SP, Fox BG (2014). Expression, purification and characterization of a functional carbohydrate-binding module from *Streptomyces* sp. SirexAA-E. Protein Expr Purif.

[75] Nishijima H (2015). Extra tyrosine in the carbohydrate-binding module of *Irpex lacteus* Xyn10B enhances its cellulose-binding ability. Biosci Biotechnol Biochem.

[76] Singh S, Simmons BA, Vogel KP (2009). Visualization of biomass solubilization and cellulose regeneration during ionic liquid pretreatment of switchgrass. Biotechnol Bioeng.

[77] Li C (2011). Influence of physico-chemical changes on enzymatic digestibility of ionic liquid and AFEX pretreated corn stover. Bioresour Technol.

[78] Petkun S (2010). Structure of a family 3b’ carbohydrate-binding module from the Cel9V glycoside hydrolase from *Clostridium thermocellum*: structural diversity and implications for carbohydrate binding. Acta Crystallogr D Biol Crystallogr.

[79] Yaniv O (2014). Fine-structural variance of family 3 carbohydrate-binding modules as extracellular biomass-sensing components of *Clostridium thermocellum* anti-sigmaI factors. Acta Crystallogr D Biol Crystallogr.

[80] Najmudin S (2006). Xyloglucan is recognized by carbohydrate-binding modules that interact with beta-glucan chains. J Biol Chem.

[81] Czjzek M (2001). The location of the ligand-binding site of carbohydrate-binding modules that have evolved from a common sequence is not conserved. J Biol Chem.

[82] Charnock SJ (2000). The X6 “thermostabilizing” domains of xylanases are carbohydrate-binding modules: structure and biochemistry of the *Clostridium thermocellum* X6b domain. Biochemistry.

[83] Shin ES (2002). Influence of the transposition of the thermostabilizing domain of *Clostridium thermocellum* xylanase (XynX) on xylan binding and thermostabilization. Appl Environ Microbiol.

[84] Najmudin S (2010). Putting an N-terminal end to the *Clostridium thermocellum* xylanase Xyn10B story: crystal structure of the CBM22-1-GH10 modules complexed with xylohexaose. J Struct Biol.

[85] von Ossowski I (2005). Protein disorder: conformational distribution of the flexible linker in a chimeric double cellulase. Biophys J.

[86] Sammond DW (2012). Cellulase linkers are optimized based on domain type and function: insights from sequence analysis, biophysical measurements, and molecular simulation. PLoS One.

[87] Gao L (2015). Linker length and flexibility induces new cellobiohydrolase activity of PoCel6A from *Penicillium oxalicum*. Biotechnol J.

[88] Batista PR (2011). High temperatures enhance cooperative motions between CBM and catalytic domains of a thermostable cellulase: mechanism insights from essential dynamics. Phys Chem Chem Phys.

[89] Weiss JN (1997). The Hill equation revisited: uses and misuses. FASEB J.

[90] Henshaw JL (2004). The family 6 carbohydrate binding module CmCBM6-2 contains two ligand-binding sites with distinct specificities. J Biol Chem.

[91] Southall SM (1999). The starch-binding domain from glucoamylase disrupts the structure of starch. FEBS Lett.

[92] Li C (2010). Comparison of dilute acid and ionic liquid pretreatment of switchgrass: biomass recalcitrance, delignification and enzymatic saccharification. Bioresour Technol.

[93] Zhang Y-HP (2006). A transition from cellulose swelling to cellulose dissolution by o-phosphoric acid: evidence from enzymatic hydrolysis and supramolecular structure. Biomacromolecules.

[94] Bansal P (2009). Modeling cellulase kinetics on lignocellulosic substrates. Biotechnol Adv.

[95] Thompson AJ (2012). Structural and mechanistic insight into N-glycan processing by endo-alpha-mannosidase. Proc Natl Acad Sci USA.

[96] UniProt C. Reorganizing the protein space at the Universal Protein Resource (UniProt). Nucleic Acids Res. 2012;40(Database issue):D71–5. 10.1093/nar/gkr981 PMC3245120 22102590

[97] Klock HE, Lesley SA (2009). The polymerase incomplete primer extension (PIPE) method applied to high-throughput cloning and site-directed mutagenesis. Methods Mol Biol.

[98] Blommel PG (2007). Enhanced bacterial protein expression during auto-induction obtained by alteration of lac repressor dosage and medium composition. Biotechnol Prog.

[99] Blommel PG, Fox BG (2007). A combined approach to improving large-scale production of tobacco etch virus protease. Protein Expr Purif.

[100] Weimer PJ, Lopez-Guisa JM, French AD (1990). Effect of cellulose fine structure on kinetics of its digestion by mixed ruminal microorganisms in vitro. Appl Environ Microbiol.

[101] Chundawat SP (2010). Multifaceted characterization of cell wall decomposition products formed during ammonia fiber expansion (AFEX) and dilute acid based pretreatments. Bioresour Technol.

[102] Kluepfel D (1990). Purification and characterization of a new xylanase (xylanase B) produced by *Streptomyces lividans* 66. Biochem J.

[103] Morais S (2012). Functional association of catalytic and ancillary modules dictates enzymatic activity in glycoside hydrolase family 43 beta-xylosidase. J Biol Chem.

[104] Sluiter A, et al. NREL analytical procedure LAP-002, determination of structural carbohydrates and lignin in biomass. Golden: National Renewable Energy Laboratory; 2004.

[105] Sluiter A, et al. LAP-005 NREL analytical procedure, determination of ash in biomass. Golden: National Renewable Energy Laboratory; 2004.

[106] Tomme P (2000). Affinity electrophoresis for the identification and characterization of soluble sugar binding by carbohydrate-binding modules. Enzyme Microb Technol.

[107] Mortz E (2001). Improved silver staining protocols for high sensitivity protein identification using matrix-assisted laser desorption/ionization-time of flight analysis. Proteomics.

[108] Miller GL (1959). Use of dinitrosalicylic acid reagent for determination of reducing sugar. Anal Chem.

[109] Nelder JA, Mead R (1965). A simplex-method for function minimization. Comput J.

