# Supplementary figures and images for: Multifunctional cellulase catalysis targeted by fusion to different carbohydrate-binding modules
Source: Biotechnol Biofuels. 2015 Dec 21;8:220. doi: 10.1186/s13068-015-0402-0 (PMC4687162; doi:10.1186/s13068-015-0402-0)

Arabinoxylan

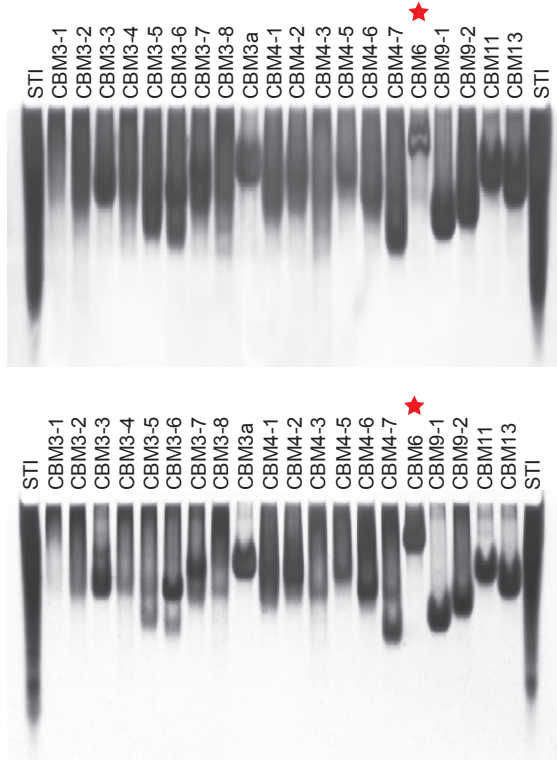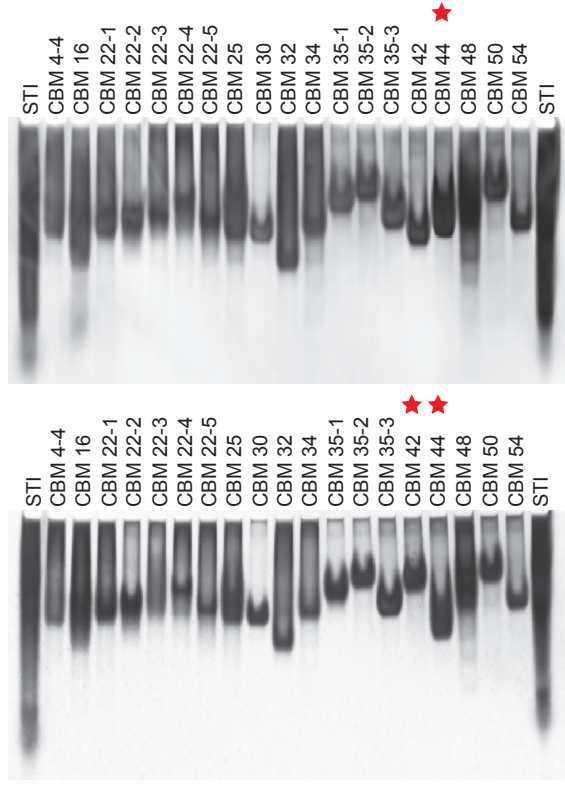

Beechwood Xylan

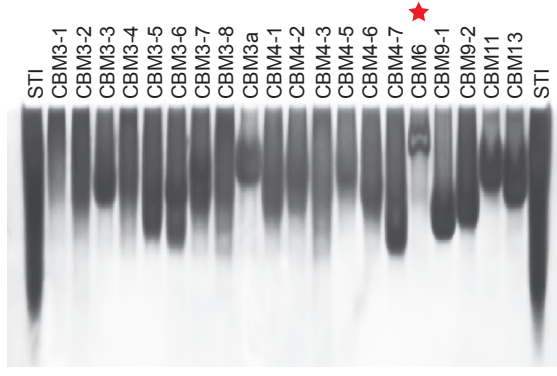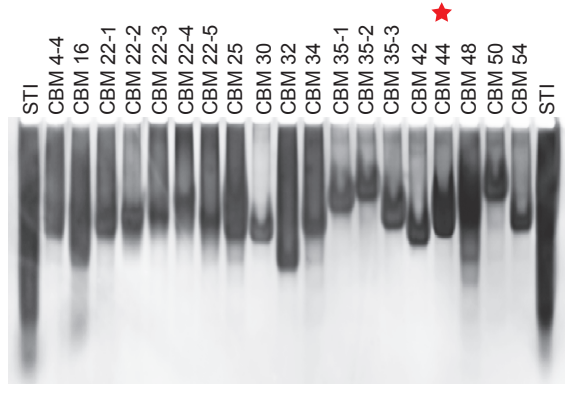

Galactomannan

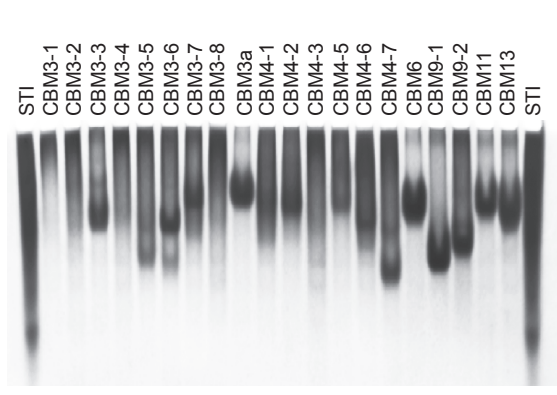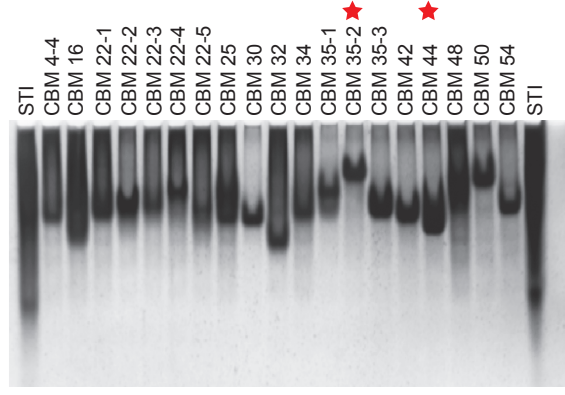

Lichenan

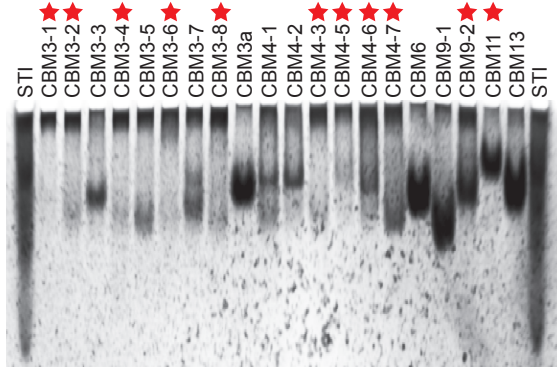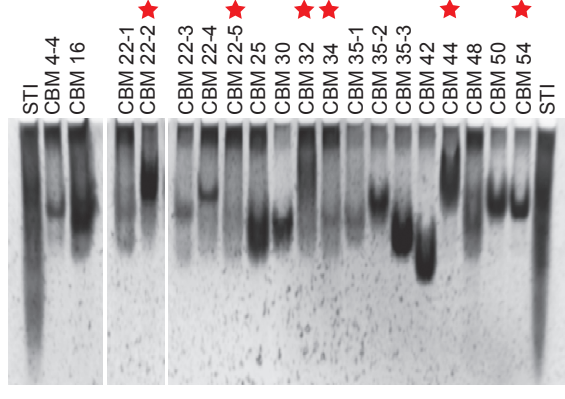

No substrate

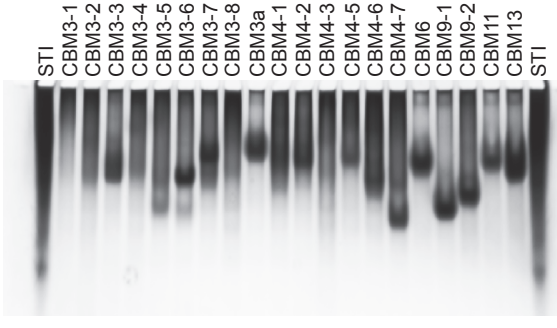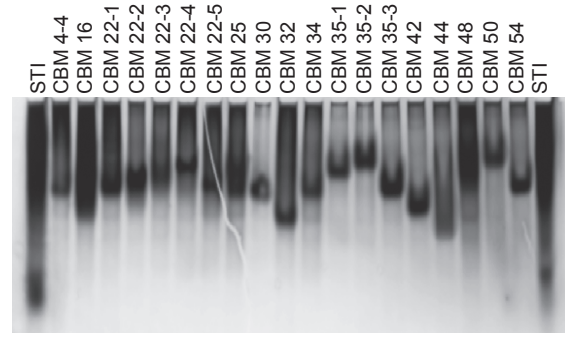

Supplement: Supplementary file 2 — 10.1186/s13068-015-0402-0 Affinity gel electrophoresis of GFP_CBM fusions. Thirty-nine GFP_CBM fusions were tested for binding specificities in native polyacrylamide gels containing CaCl2 and either lichenan, galactomannan, beechwood xylan, or arabinoxylan. A “No substrate” gel is shown for comparison with the substrate gels. Red stars indicate where binding was detected. Soybean trypsin inhibitor (STI) was used as a control for no binding. [file 13068_2015_402_MOESM2_ESM.pdf]
