# Supplementary material for: Multifunctional cellulase catalysis targeted by fusion to different carbohydrate-binding modules
Source: Biotechnol Biofuels. 2015 Dec 21;8:220. doi: 10.1186/s13068-015-0402-0 (PMC4687162; doi:10.1186/s13068-015-0402-0)
Supplement: Supplementary file 3 — 10.1186/s13068-015-0402-0 Differential equations corresponding to the kinetic schemes of Figure 7 are presented in Mathematica format. [file 13068_2015_402_MOESM3_ESM.docx]

Differential equations corresponding to the kinetic scheme shown in Figure 7A for reaction of cellulose.

y[1] = cellulose

y[2] = glucose

y[3] = cellobiose

y[4] = cellotriose

y[5] = cellotetraose

dy[1]/d[t] = -(k1 + k2 + k3 + k4 ) y[1][t]

dy[2]/d[t] = (k1) y[1][t] + 2 k5 y[3][t] + k6 y[4][t] + k7 y[5][t]

dy[3]/d[t] = (k2) y[1][t] + k6 y[4][t] + 2 k8 y[5][t] - k5 y[3][t]

dy[4]/d[t] = (k3) y[1][t] + k7 y[5][t] - k6 y[4][t]

dy[5]/d[t] = (k4) y[1][t] - k7 y[5][t] - k8 y[5][t]

Differential equations corresponding to the kinetic scheme shown in Figure 7B for reaction of hemicellulose.

y[1] = hemicellulose

y[2] = pentose

y[3] = pentobiose

y[4] = pentotriose

y[5] = pentotetraose

y[6] = pentopentaose

dy[1]/d[t] = -(k1 + k2 + k3 + k4 + k5) y[1][t]

dy[2]/d[t] = (k1) y[1][t] + 2 k6 y[3][t] + k7 y[4][t] + k8 y[5][t] + k9 y[6][t]

dy[3]/d[t] = (k2) y[1][t] +k7 y[4][t] + 2 k10 y[5][t] + k11 y[6][t] - k6 y[3][t]

dy[4]/d[t] = (k3) y[1][t] + k8 y[5][t] + k11 y[6][t] - k7 y[4][t]

dy[5]/d[t] = (k4) y[1][t] + k9 y[6][t] - k8 y[5][t] - k10y[5][t]

dy[6]/d[t] = (k5) y[1][t] - k9 y[6][t] - k11 y[6][t]
